# Detection and characterization of microRNA expression profiling and its target genes in response to canine parvovirus in Crandell Reese Feline Kidney cells

**DOI:** 10.7717/peerj.8522

**Published:** 2020-02-12

**Authors:** Phongsakorn Chuammitri, Soulasack Vannamahaxay, Benjaporn Sornpet, Kidsadagon Pringproa, Prapas Patchanee

**Affiliations:** 1 Department of Veterinary Biosciences and Public Health, Faculty of Veterinary Medicine, Chiang Mai University, Chiang Mai, Thailand; 2 Center of Excellence in Veterinary Biosciences (CEVB), Chiang Mai University, Chiang Mai, Thailand; 3 Central Laboratory, Faculty of Veterinary Medicine, Chiang Mai University, Chiang Mai, Thailand; 4 Department of Food Animal Clinics, Faculty of Veterinary Medicine, Chiang Mai University, Chiang Mai, Thailand; 5 Integrative Research Center for Veterinary Preventive Medicine, Chiang Mai University, Chiang Mai, Thailand

**Keywords:** MicroRNAs, Canine parvovirus, CPV-2c, miR-1247-3p, Target genes, Crandell Reese Feline Kidney cells, Small RNA-seq

## Abstract

**Background:**

MicroRNAs (miRNAs) play an essential role in gene regulators in many biological and molecular phenomena. Unraveling the involvement of miRNA as a key cellular factor during in vitro canine parvovirus (CPV) infection may facilitate the discovery of potential intervention candidates. However, the examination of miRNA expression profiles in CPV in tissue culture systems has not been fully elucidated.

**Method:**

In the present study, we utilized high-throughput small RNA-seq (sRNA-seq) technology to investigate the altered miRNA profiling in miRNA libraries from uninfected (Control) and CPV-2c infected Crandell Reese Feline Kidney cells.

**Results:**

We identified five of known miRNAs (miR-222-5p, miR-365-2-5p, miR-1247-3p, miR-322-5p and miR-361-3p) and three novel miRNAs (Novel 137, Novel 141 and Novel 102) by sRNA-seq with differentially expressed genes in the miRNA repertoire of CPV-infected cells over control. We further predicted the potential target genes of the aforementioned miRNAs using sequence homology algorithms. Notably, the targets of miR-1247-3p exhibited a potential function associated with cellular defense and humoral response to CPV. To extend the probing scheme for gene targets of miR-1247-3p, we explored and performed Gene Ontology (GO) enrichment analysis of its target genes. We discovered 229 putative targets from a total of 38 enriched GO terms. The top over-represented GO enrichment in biological process were lymphocyte activation and differentiation, marginal zone B cell differentiation, negative regulation of cytokine production, negative regulation of programed cell death, and negative regulation of signaling. We next constructed a GO biological process network composed of 28 target genes of miR-1247-3p, of which, some genes, namely *BCL6*, *DLL1*, *GATA3*, *IL6*, *LEF1*, *LFNG* and *WNT1* were among the genes with obviously intersected in multiple GO terms.

**Conclusion:**

The miRNA-1247-3p and its cognate target genes suggested their great potential as novel therapeutic targets or diagnostic biomarkers of CPV or other related viruses.

## Introduction

Canine parvovirus (CPV) is a common cause of viral enteritis in puppies with high morbidity and mortality. The virus has a wide host range in many mammalian families, including dogs, cats, ferrets, minks and raccoons (Kapil et al., 2007). CPV is a non-enveloped virus, single-stranded DNA virus in the *Parvoviridae* family (Cotmore et al., 2014). The compact genome (approximately 5,000 nt) encodes two nonstructural proteins (NS1 and NS2) and three capsid proteins (VP1, VP2 and VP3) through an alternative splicing of the same mRNAs (Berns & Parrish, 2007). CPV is globally distributed, and this virus has been evolutionarily diverged from CPV-1 to its present forms of CPV-2 subtypes; CPV-2, CPV-2a, CPV-2b and CPV-2c (Truyen, 2006). CPV-2 can cause severe hemorrhagic gastroenteritis in dogs, owing to amino acid substitutions harboring at VP2 capsid protein (Truyen, 2006).

MicroRNAs (miRNAs) are small non-coding RNAs, produced within the nuclei from miRNA gene transcription. The miRNA biogenesis starts with a long nucleotide sequences of the miRNAs which are folded into hairpin configuration, known as “primary miRNAs or pri-miRNAs”. Later on, the shortening and editing of pri-miRNAs occurred by the activity of Drosha and DGCR9 enzymes, making a shorter version of miRNAs and soon to be called “pre-miRNA”. The pre-miRNA, an ~70-nucleotide in length, is exported to the cytosol where the Dicer repeatedly chops the sequences once again here to make an ~20-bp miRNA/miRNA* duplex (Krol, Loedige & Filipowicz, 2010). The functional miRNA, ~18–22 nt long, represents a mature miRNA, which can be incorporated into the miRNA-induced silencing complex (miRISC). The miRNAs, altogether with its cognate target mRNAs, function as gene expression regulators as part of miRISC by preventing protein translation, mRNA deadenylation, and mRNA target cleavage (Krol, Loedige & Filipowicz, 2010).

MicroRNAs play a pivotal role in many biological and molecular phenomena. Some miRNAs are involved in the regulation of genes related to the specific process in mammalian cells, such as cell differentiation, cell migration, cell signaling, various pathologies and apoptosis (Bartel, 2004; Krol, Loedige & Filipowicz, 2010). Some particular miRNAs may have a universal function during virus establishment and infections. Understanding the involvement of host factors in virus infection, modulated by miRNAs, may facilitate the discovery of potential intervention candidates, that is, therapeutic silencing of miR-122 in chronic hepatitis C virus infection (Henke et al., 2008). The potential targets of conserved and novel miRNAs could be predicted using sequence homology algorithms across different mammalian species. The deregulation of miRNA can involve in tumorigenesis, and some miRNAs are known to behave as an oncogene or tumor suppressor genes (Bartel, 2004). Furthermore, miRNAs have great potential as diagnostic or prognostic biomarkers and novel therapeutic targets. However, the change of miRNA expression profiles in CPV in tissue culture systems has not been fully elucidated.

Recently, a few researchers have studied the miRNA repertoire using domestic cats (*Felis catus*) or cell culture originated from feline kidney cells as a basis to study CPV/Mink enteritis virus (MEV) infection model (Sun et al., 2014; Zhou et al., 2017). In the present study, we performed and utilized high throughput approach via miRNA-seq experiments to identify the altered miRNA profiling in miRNA libraries of uninfected and CPV-2c infected Crandell Reese Feline Kidney (CRFK) cells. Among miRNA repertoire retrieved from sequencing libraries, we would expect to see a dramatic expression of many miRNAs, but interestingly, miR-1247-3p appears to be one associated with cellular and humoral host defense response to CPV. At this point, there is little or nothing known about miR-1247 regarding its functionality related to CPV or other viruses. In this report, we have identified the targets of miR-1247-3p, along with other miRNAs; miR-222-5p, miR-365-2-5p, miR-322-5p and miR-361-3p, in CRFK cell infected with CPV-2c. We also analyze and report on relevant data between miR-1247-3p and its essential target genes representing biological pathways associated with CPV infection.

## Materials and Methods

### Clinical and viral samples

Rectal swab samples of four dogs with clinical signs of hemorrhagic gastroenteritis and suspected of CPV infection were analyzed. Samples were collected when the outbreak of CPV had occurred in Vientiane, Laos, during February–April, 2016 (Vannamahaxay et al., 2017).

### Ethics statement and sample collection

Animal use protocol for taking rectal swab samples from dogs was approved by the Institutional Animal Care and Use Committee, Faculty of Veterinary Medicine, Chiang Mai University, Thailand (FVM-ACUC, Permission No. R14/2559). Written informed consent was provided by each dog’s owner. Cotton swabs soaked with sterile phosphate-buffered saline containing antibiotics were used. Rectal swab samples were kept frozen for further analysis of CPV by PCR/real-time PCR and virus isolation.

### Cell culture and maintenance

Crandell Reese Feline Kidney cells (CRFK ATCC^®^ CCL-94™, Lot: 60980363) derived from *F. catus* (cat) cortex of kidney at passage no. 182 were propagated and maintained in 25 cm^2^ flasks filled with Dulbecco’s Modified Eagle Medium high glucose (DMEM, Gibco; Thermo Fisher Scientific, Waltham, MA, USA) supplemented with 5% v/v fetal bovine serum, 200 IU/ml penicillin G Sodium/200 μg/mL Streptomycin sulfate (Gibco). Cells were incubated at 37 °C in a humidified 5% CO_2_ incubator until 80% confluent monolayer before further experiments (Crandell, Fabricant & Nelson-Rees, 1973; Luo et al., 2016).

### Virus isolation and harvest

Fecal samples containing defined CPV-2c strains (Accession # KX601665 to KX60168) were thawed at RT; homogenized completely; spun at 8,000 rpm for 5 min at RT. The supernatant was aspirated and filtered through 0.22 μm syringe filter (Minisart^®^ Syringe Filter, Sartorius, Goettingen, Germany). Filtered fluid containing the virus was kept frozen (−80 °C) until use.

Confluent CRFK monolayer cells (80%) in 25 cm^2^ flasks were infected with supernatant as stated earlier. After adsorbed for 1 h at 37 °C, the inoculum was removed, and DMEM with 5% FBS was added then the cells were again incubated at 37 °C. Cell cultures were observed daily during 3–5 days to monitor the appearance of cytopathic effects (CPE). Sample exhibited CPE in the infection conditions with some forms of rounding and clumping within 48 h and detachment within 72 h of incubation. The infected CRFKs were subjected to three rounds of freezing and thawing cycles. The supernatants were collected; tested for genomic DNA of CPV; stored at −80 °C until further use.

### Infection of CRFK cells

Viral samples were flash thawed at 37 °C by submerging in water bath. The cryotubes containing thawed virus were spun at 8,000 rpm for 5 min at 4 °C. The supernatant was aspirated and filtered through 0.22 μm syringe filter. Confluent CRFK monolayer cells (80%) in 25 cm^2^ flasks were infected with CPV-2c strains at MOI of 0.1. After adsorbed for 1 h at 37 °C, the inoculum was removed, and DMEM with 5% FBS was added then the cells were again incubated at 37 °C. Cell cultures were observed daily during 3–5 days to monitor the appearance of CPE. Samples appeared with CPE, no later than 96 h were subjected to RNA isolations.

### RNA isolation, library preparation and sequencing

#### RNA isolation

Total RNA of CRFK infected with CPV-2c Laotian stains (CPV) or control was isolated using RNAzol^®^ RT reagent (Sigma–Aldrich, St. Louis, MO, USA) plus Direct-zol™ RNA Miniprep kit (Zymo Research, Irvine, CA, USA) as manufacture’s protocol with slight modifications. In brief, the CRFK cells were lysed directly on cell culture flask with RNAzol RT reagent and mixed thoroughly. Then, the sample lysates were proceeded to RNA purification using Zymo-Spin™ IIC Column. The total RNA were eluted with DNase/RNase-free water.

#### RNA quantification and qualification

The 1% agarose gels were run to check the RNA degradation/contamination. RNA purity was verified using the NanoPhotometer^®^ spectrophotometer (Implen, Inc., Westlake Village, CA, USA) and performed by Novogene Experimental Department (Novogene Genomics, Singapore). RNA integrity together with quantitation were evaluated with RNA Nano 6000 Assay Kit of the Agilent Bioanalyzer 2100 system (Agilent Technologies, Santa Clara, CA, USA).

#### Library preparation for clustering and small RNA sequencing

A total amount of three μg total RNA per sample was aliquoted and later prepared a small RNA (sRNA) library. For sequencing library generation, NEBNext^®^ Multiplex sRNA Library Prep Set for Illumina^®^ (New England Biolabs, Ipswich, MA, USA) was used as per manufacturer’s directions. The adding of index codes was done to assign sequences for each sample performed. TruSeq SR Cluster Kit v3-cBot-HS (Illumina, San Diego, CA, USA) was used to performing the clustering of index-coded samples. The libraries were sequenced on an IlluminaHiseq 2000 machine to generate the 50 bp single-end reads. The whole process of sequencing was performed at Novogene.

### MicroRNA expression analyses

#### Quality control and reads mapping to the reference sequence

Raw reads of *fastq* format were processed and filtered through *perl* and *python* scripts. The high-quality reads were obtained with Q20, Q30 margin from the calculation. The downstream analyses were based mainly on the clean and high-quality reads. The Fastq sequence of sRNA reads was mapped to reference genome (*Mus musculus* mm10) by Bowtie (Langmead et al., 2009).

#### Known miRNA alignment

The miRBase release 22.1 (October 2018) was used as reference. The miRDeep2 (Friedländer et al., 2011) was served as a tool for searching for known miRNA and sRNA-tools-cli helped identify the potential miRNA. The miRNA counts and base bias on the first position of miRNA were obtained using custom scripts in *perl*. RepeatMasker, Rfam database or annotations compiled from other similar databases were used as a tool to identify and remove sequences from protein-coding genes, repeat sequences, rRNA, tRNA, snRNA and snoRNA. The remaining unique sRNA sequences were kept for further analysis.

#### Novel miRNA prediction

The miREvo (Wen et al., 2012) and miRDeep2 softwares (Friedländer et al., 2011) were employed to predict novel miRNA from un-annotation in the previous steps, as well as, helped identify novel miRNA counts, base bias on either the first position or on each position of all novel miRNAs.

#### Small RNA annotation

The unique sRNA (mapped to only one annotation) was allocated using this priority rule: known miRNA > rRNA > tRNA > snRNA > snoRNA > repeat > gene > NAT-siRNA > gene > novel miRNA > ta-siRNA.

#### miRNA editing analysis

The seed region, position 2–8 of a mature miRNA, was identified by aligning all sRNA tags to mature miRNA. Only one nucleotide mismatch at the seed region was permitted.

#### miRNA family analysis

The miFam.dat (http://www.mirbase.org/ftp.shtml) was used to explore the miRNA families in our samples. The Rfam (http://rfam.sanger.ac.uk/search/) aided in searching for novel miRNA precursor family.

#### Target gene prediction

The miRNA target gene prediction was executed by using miRanda (Enright et al., 2003).

### Quantification of miRNA

The miRNA read counts was normalized as transcript per million (TPM) (Zhou et al., 2010) based on the following formula: Normalized expression = mapped read count/Total reads × 10^6^.

### Differential expression of miRNA

The DESeq R package (1.20.0) (Anders & Huber, 2010; Wang et al., 2010) and iDEP.90 (http://bioinformatics.sdstate.edu/idep/) (Ge, Son & Yao, 2018) were facilitated differential gene expression analysis. The resulting *p*-values were adjusted using the Benjamini and Hochberg’s false discovery rate (FDR) (Benjamini & Hochberg, 1995). Genes with an adjusted *p*-value < 0.05 found by DESeq2 were assigned as differentially expressed. The *p*-value was adjusted using *q* value (Storey, 2003). The *q* value < 0.05 and log2 (fold change) > 1 was set as the threshold for significant differential expression by default.

### Gene ontology and KEGG enrichment analysis of differentially expressed miRNA genes

Gene Ontology (GO) enrichment analysis of differentially expressed miRNA genes were implemented by the iDEP.90 web-based analysis software, PANTHER GO Enrichment Analysis (http://geneontology.org/) (Ashburner et al., 2000; The Gene Ontology Consortium, 2019; Mi, Muruganujan & Thomas, 2012), g:Profiler/g:GOSt/g:Convert (https://biit.cs.ut.ee/gprofiler/gost) (Raudvere et al., 2019; Reimand et al., 2007), ClueGO and CluePedia Cytoscape plug-in (http://apps.cytoscape.org/apps/cluego) (Bindea et al., 2009) and GOseq R package, in which gene length bias was corrected. GO terms with corrected *p*-value less than 0.05 were considered significantly enriched by differential expressed genes (Young et al., 2010). Kyoto Encyclopedia of Genes and Genomes, KEGG (https://www.genome.jp/kegg/) and KOBAS were used as a resource for examining the pathways and associated functions of genes (Kanehisa et al., 2007; Mao et al., 2005; Kanehisa & Goto, 2000).

### Protein–Protein interaction analysis of differentially expressed genes

Protein–protein interaction (PPI) analysis of differentially expressed genes (DEGs) were based on feline species (*F. catus*) in the STRING database (https://string-db.org/) (Szklarczyk et al., 2018).

### Statistical analysis of miRNA-seq experiments

Statistical analyses were performed using the R software version 3.5.1/R studio version 1.1.456. The graphic illustrations were also generated by VennDiagram, calibrate, datasets, ggplot2, viridis R packages. The coefficient of determination (*R*^2^) and *Z*-score were computed by *R* and the heatmap.2 function of the gplots R package. Differential expression analysis was carried out using DESeq2. Chord diagram pairwise correlations between clusters were visualized as chord diagrams in R using the circlize package. For dimensionality reduction visualizations, we used the *t*-SNE algorithm (Van der Maaten & Hinton, 2008). FDR was used for *p*-value correction (Benjamini & Hochberg, 1995). The *p-*value of < 0.05 considered to be statistically significant.

## Results

### Summary of sRNA library dataset by deep sequencing in CRFK cells infected with CPV-2c Laotian isolates

We have constructed two libraries from uninfected cells (Control) and CPV-2c infections (CPV) to analyze the miRNA collections. To identify the target genes, Illumina HiSeq 2000 was utilized. A total of raw reads ranged from 23,466,804 to 26,337,331 reads were obtained, of which 25,230,038 and 25,410,594 raw reads were generated in control cells sRNA libraries, and 23,466,804 and 26,337,331 were produced in CPV-2 infected cells, respectively (Table S1).

**Figure 1:**
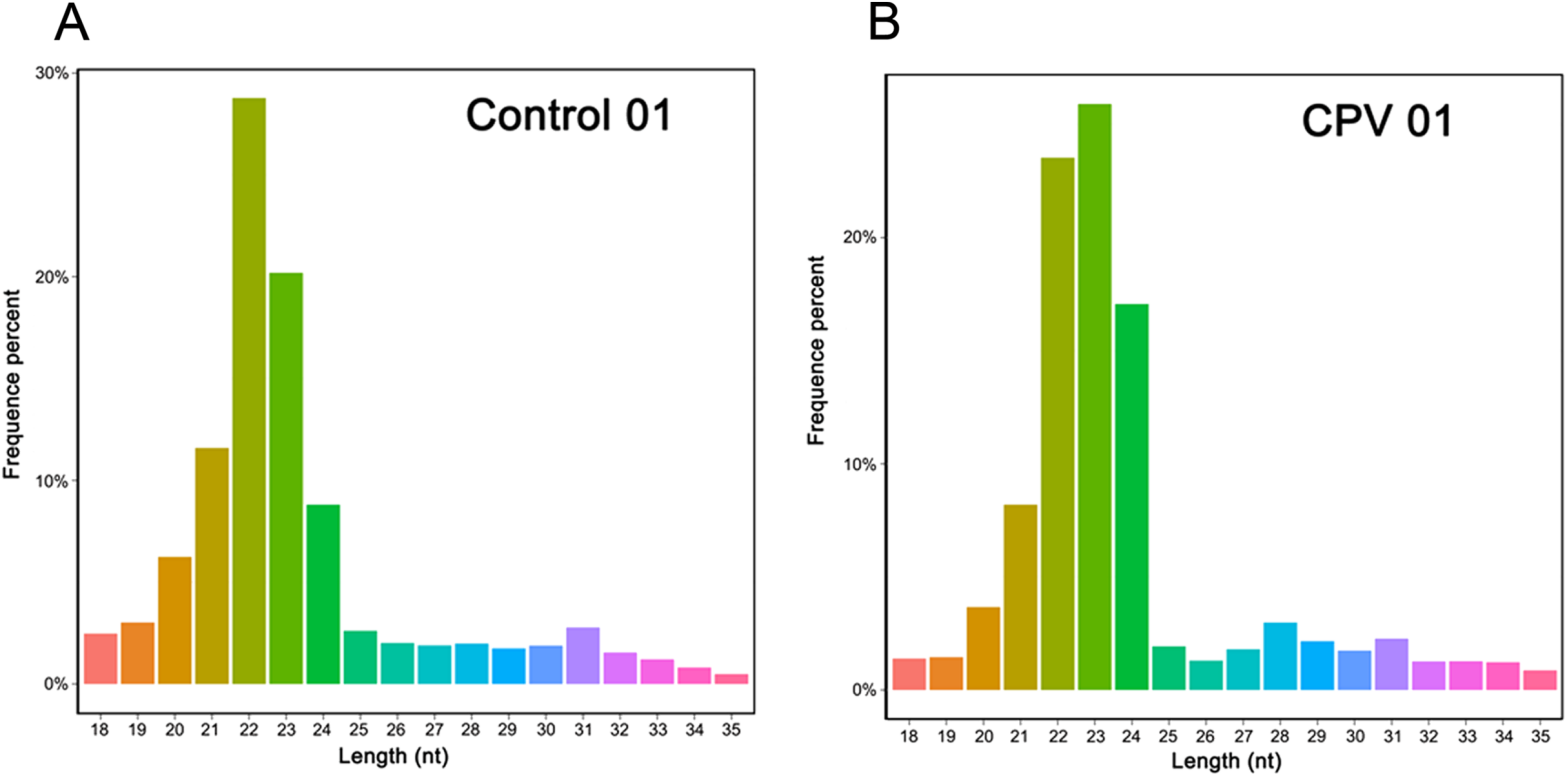
Length distribution of small RNAs in the Control (A) and CPV infection (B) libraries of CRFK cells. *x*-axis, length of small RNA distribution; *y*-axis, percentage frequency of corresponding raw reads (representative figures).

Moreover, 98.66% and 98.08% raw reads of high quality for the Control, and 97.63% and 98.48% libraries for CPV groups were defined as clean reads, respectively. For further length distribution analysis, 18–30 nucleotides (nt) total sRNA sequences were selected (Fig. 1), with 21–22 nt long sequences with high frequency is routinely considered the length of miRNAs in animals. The frequency of Control sRNA libraries was 28.33% and 20.85% (22 nt), while the CPV sRNA libraries was 25.91% (23 nt) and 30.29% (22 nt). The percentage of sRNAs of 24 nt was significantly higher than that of other sRNAs.

Next, we performed the mapping of the sRNA reads to the genome by bowtie to analyze their expression and distribution in the genome. Based on unavailable database of miRNA in *F. catu*s genome, the sRNAs were allocated against the mouse genome. A total of 17,464,428–17,492,270 reads (76.92–78.48%) unique mapped sRNA was found in Control libraries, and 15,118,021–19,220,070 reads (70.39–76.74%) mapped sRNA was indicated in CPV libraries, respectively. These mapped sRNAs in each library comprised of known miRNAs, other RNA species, including putative novel miRNAs, and unannotated other fragments (Table S2). Additionally, unannotated reads accounted for 4.51–8.68% in all libraries (Table S2).

### Analysis of known miRNA and identification of conserved and evolutionarily known miRNAs

**Figure 2:**
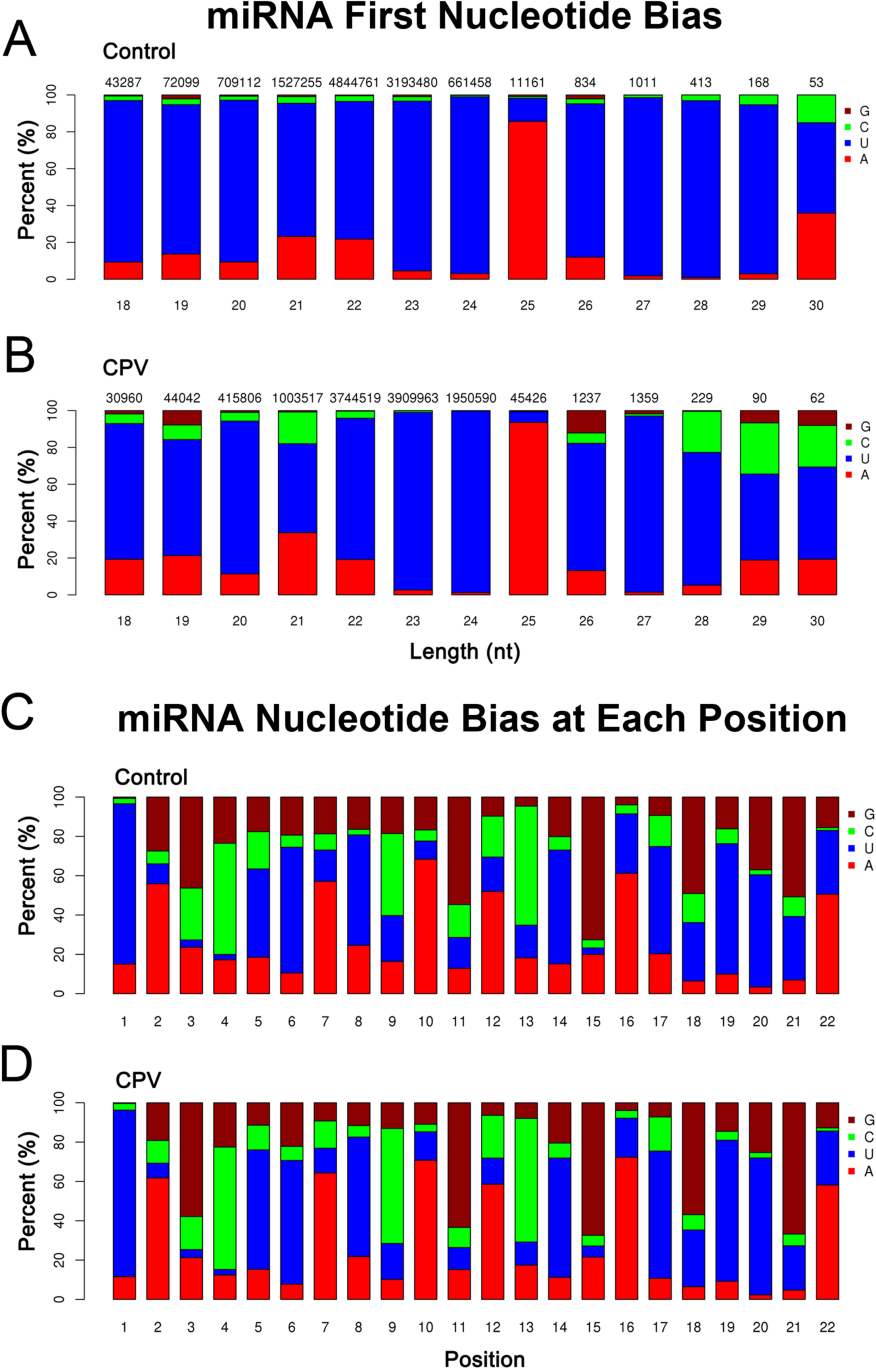
First nucleotide bias and nucleotide bias at each position of known feline miRNAs. (A) First nucleotide bias of known *Felis catus* miRNA candidates in control and (B) CPV libraries. The number on top of the bars indicated the number of sequences corresponding to the miRNA length (nt). *x*-axis, length of miRNAs; *y*-axis, responding percentages of each nucleotide. (C) Nucleotide bias at each position in control and (D) CPV libraries. *x*-axis, position of nucleotide in miRNAs; *y*-axis, responding percentages of each nucleotide.

miRNA is produced by Dicer from pri-miRNA. Because of specificity of the cleavage site, miRNA has some preference on bases at different positions. For example, the first base from the 5′ end has a strong preference of U (Fig. 2A), but resistant to G; bases from position 2 to 4 on the 5′ end are usually resistant to U; bases from position 10 (this position is the cleavage site when miRNA regulates mRNA) has a strong A preference (Fig. 2B). The mapped reads align to a specific special in the miRBase22 (v22.1, October 2018) get the details of each sample’s known miRNA information. The majority of the first base from the 5′ end of mature miRNAs had U (21–24 nt). At bases 2–4, the resistance to U was indicated. At base 10, the strong preference of A was located (Fig. 2B). These findings indicated the specificity of miRNAs found in both Control and CPV infected cells, as reported in a theoretical way (Table S3). The miRNA information in this study was in agreement with those reports (Khaldun et al., 2015; Liu et al., 2017).

The sRNA libraries (miRNA) were searched for known mature *mmu* miRNAs that were publicly reported in miRBase22. After searching and subsequent sequence analyses, 333 known hairpin structures were revealed, which actually corresponded to 420 unique mature miRNA sequences (Table S4). Those miRNAs were conserved among animals and plant species, representing 177 diversified miRNA families. In addition, among these 420 known miRNAs, 11 miRNAs were differentially expressed in the CPV vs. Control libraries.

### Prediction of potentially novel miRNAs and nucleotide bias

The characteristic hairpin structure of miRNA precursor can be used to predict novel miRNA. We used miREvo (Wen et al., 2012) and miRDeep2 (Friedländer et al., 2011) software to predict novel miRNA. We followed the following priority rule: known miRNA > rRNA > tRNA > snRNA > snoRNA > repeat > gene > novel miRNA. The number of novel miRNAs was summarized in Table S5. The novel *F. catus* miRNAs found in this study was as follows; mapped hairpin (*n* = 141), mapped mature miRNA (*n* = 140), and mapped star (*) of miRNA (*n* = 68), respectively. The majority of these novel miRNAs had a length of 22–23 nt in both of the libraries (Control vs. CPV), and started with a 5′ U. Similarly, the majority of known miRNAs had 22–23 nt in both Control and CPV libraries, with a 5′ U. A total of 140 novel mature miRNA sequences was obtained from both Control and CPV libraries (Table S5). The novel miRNA precursors expanded from 46 to 295 nts in length. The majority of the first nucleotide bias of novel miRNAs (Fig. S1) had similar patterns as of those found in known miRNAs, as above mentioned in this section (Fig. 2).

### miRNA family analysis

We have also explored the occurrence of known miRNA and novel miRNA families identified from the sample in human, other animal species, worm, fly, fish, amphibian and plant. From our data, the member numbers of different miRNA families were 177 families listed in Table S6.

### Differentially expressed known and novel miRNAs between control and CPV infection

#### sRNA-seq correlation and characteristics of sRNA-seq data

**Figure 3:**
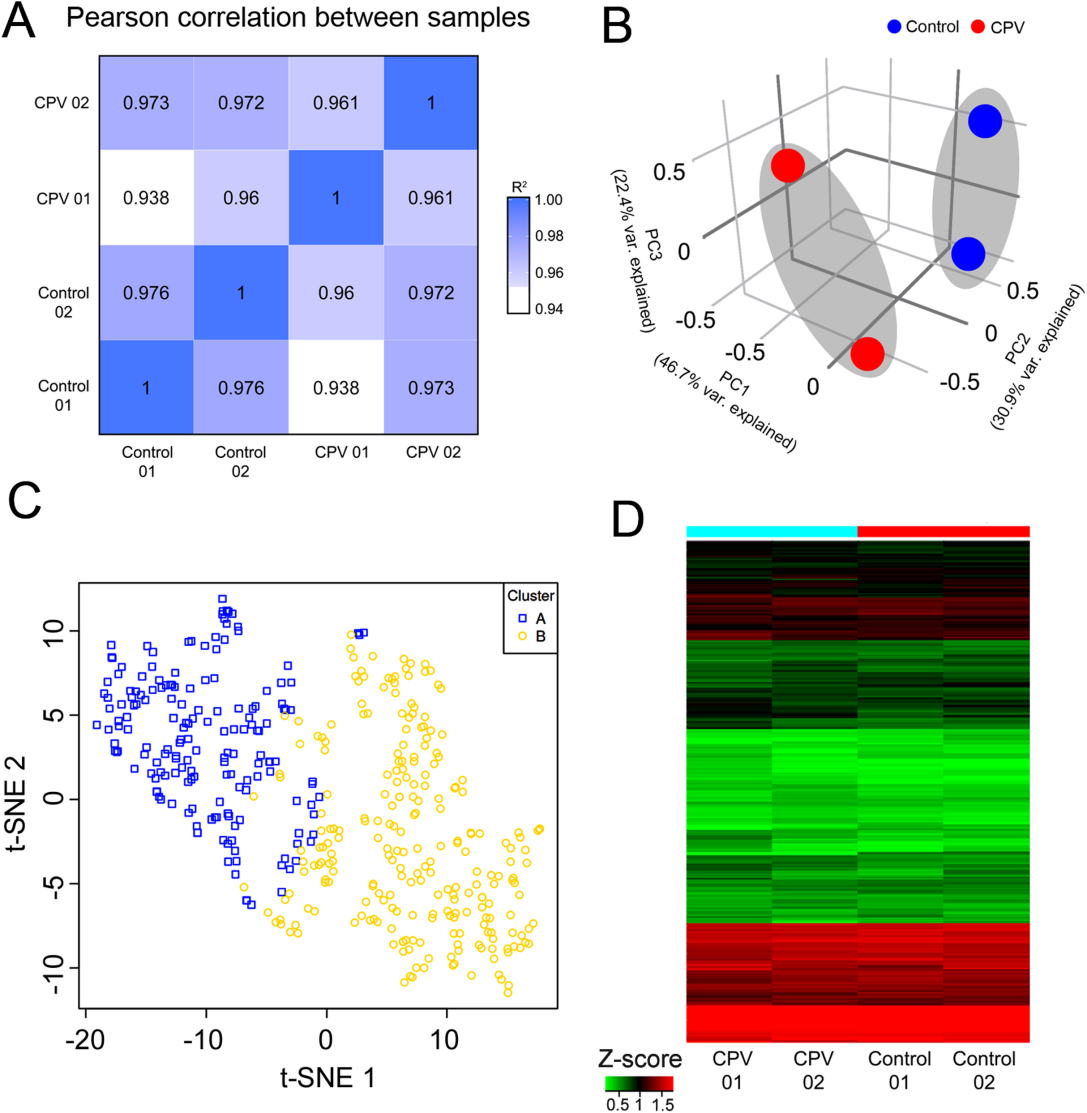
Small RNA-seq correlation and characteristics of small RNA-seq data. (A) RNA-seq correlation coefficient between each sample showed in heatmap of the Pearson correlation coefficients. The *R*^2^ across all samples was 0.938 to 0.976. (B) Principal component analysis (PCA) of gene expression profiles of all samples. Control CRFK cells (Control) are shown in blue dots; CPV infected group (CPV) presented by red dots. (C) The dimensionality reduction technique (t-SNE) of small RNA-seq data were arranged in two clusters (A and B) based on the similarity of their gene expression profiles. (D) Heatmap of gene expression (*Z*-score) in each control sample (Control) and CPV-infected sample (CPV), colors indicate the up- (red) and down-regulation (green) of gene expression.

Biological replicates are necessary for any biological experiment, including those involving RNA-seq technology (Hansen et al., 2011). In RNA-seq, replicates have two main advantages. First, they demonstrate whether the experiment is repeatable, and secondly, they can reveal differences in gene expression between samples. The correlation between samples is an important indicator for testing the reliability of the experiment. The closer the correlation coefficient is to 1, the greater the similarity of the samples. ENCODE suggests that the square of the Pearson correlation coefficient should be larger than 0.92, under ideal experimental conditions (ENCODE Project Consortium, 2004). In this project, the *R*^2^ were larger than 0.93 (Fig. 3A). Variations among technical replicates were minimal. These observations can also be confirmed by the correlation matrix. For Pearson correlation, the correlation coefficient within group and between groups were larger than 0.92 demonstrating that the experiment was repeatable, and could be revealed differences in gene expression between samples (Fig. 3A).

The Principal Component Analysis (PCA) showed that, either Control or CPV library was grouped together, differed apart from each other (Fig. 3B). PCA plot using the first and second principal components is shown in Fig. 3B. There was a clear difference between the CPV-infected and the control samples, along the first two principal components that explain 77.6% of the variance. The mixed gene expression pattern in exploratory miRNA species heterogeneity in Control and CPV infection was retrieved from sRNA-seq. To reduce the complexity of data for interpretation (*n* = 420, mapped mature miRNAs), we have performed and used the dimension reduction algorithm t-Distributed Stochastic Neighbor Embedding (t-SNE) to review our miRNA expression data (Fig. 3C). Because of high-dimensional datasets, this technique can be implemented, allowing it to be applied to large real-world datasets, resulting in well suited for the easy visualization. To identify the read count subset defined by specific miRNA gene expression signatures, unsupervised clustering of 420 mature miRNAs were identified and clustered into two groups by t-SNE, matching the known heterogeneity of transcripts of miRNA genes. The t-SNE visualization of Control/CPV showed limited overlap between two miRNA libraries. The t-SNE result of a mixture of miRNAs in Control in combination with infected-cells (CPV) showed dividing data into 2 clusters, as depicted by dots with different color schemes (cluster A–B, Fig. 3C). For heatmap and hierarchical clustering, the Control and CPV-infected cells could be customarily segmented into visibly two patterns of detection, up- and down-regulated of miRNA genes, with the broad range of uses. Four categorized groups were determined by the representation of heatmap based mainly on *Z*-score; namely from high (red) to low (green) or base-line expression (black or mixed colored), respectively (Fig. 3D).

#### miRNA expression and differential expression

**Figure 4:**
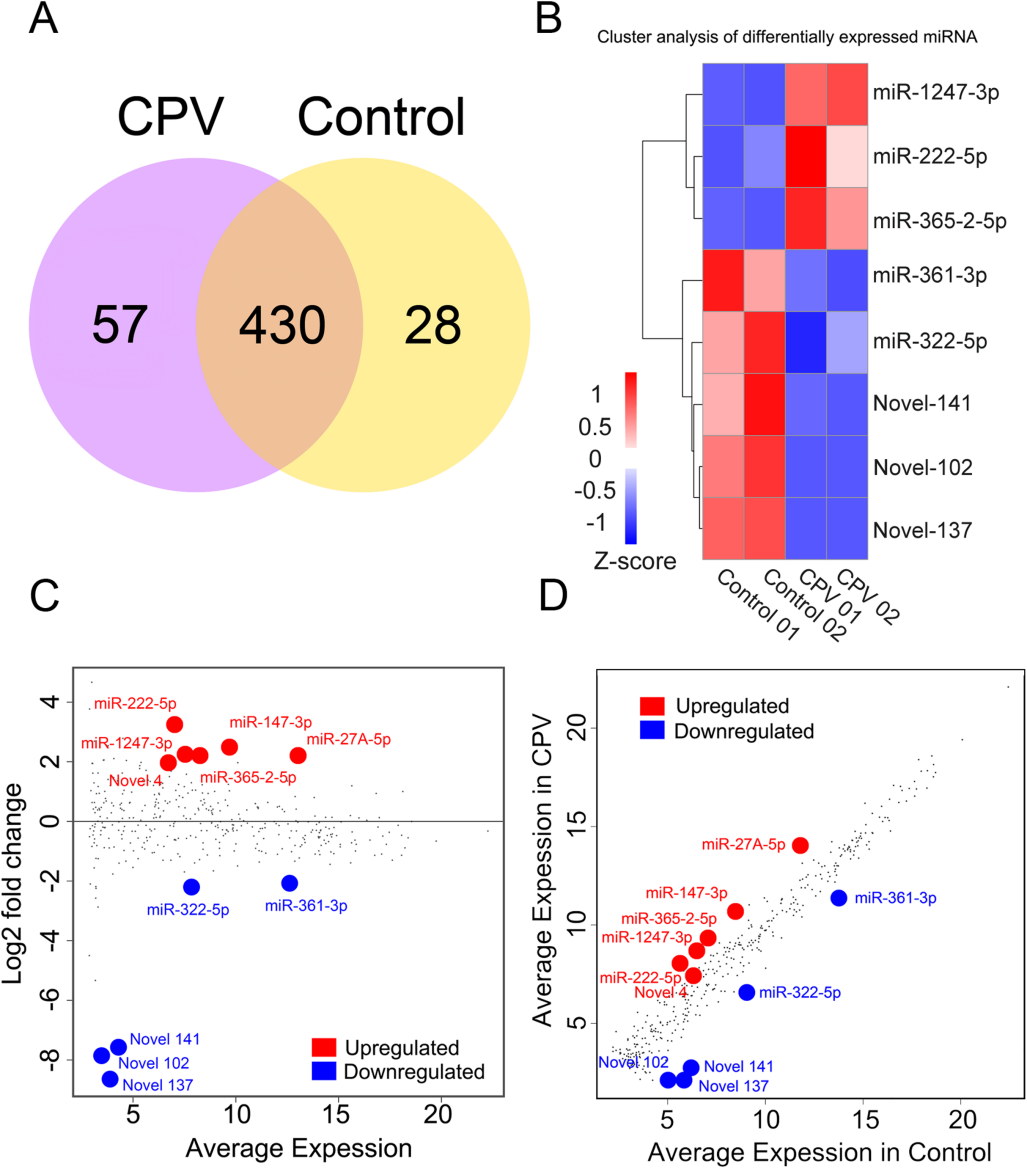
miRNA expression and differential expression. (A) Venn diagram defines the differentially expressed genes (DEGs). The numbers in each circle are the total number of genes expressed within a sample, and the overlap (*n* = 430) represents the genes expressed in common between samples. (B) Heatmap of differential gene expression (DEG) in each control sample (Control) and CPV-infected sample (CPV), colors indicate the up- (red) and down-regulation (light blue) of gene expression. (C) MA plot shows log2 fold change (*y*-axis) and the average expression (*x*-axis) across all samples. Significant changes of genes (>1 or <−1 log2 fold change) from DESeq2 (*p* < 0.05) are color highlighted. Red represents upregulation and blue are downregulated genes, gray dots are no change. (D) Scatterplot of all differentially expressed genes (DEGs) between the Control and CPV infected group by transcripts per million (TPM) reads are shown in red if they are significant upregulated, or blue when significant downregulation was found (FDR < 0.05 and fold-change > 2). Gray points are no change in gene expression.

The input data was the read count value of the miRNA expression level analysis. For samples with biological replicates, DESeq2 (Love, Huber & Anders, 2014) was used to do the analysis. To determine miRNA species from two experimental groups, each miRNA read count from Control and CPV group were compared by the transcripts per million (TPM) (Zhou et al., 2010). The Venn diagram (Fig. 4A) presents the number of miRNAs that were uniquely expressed within each group, with the overlapping regions showing the number of miRNAs that were expressed in two groups. The number of expressed miRNAs differed between the CPV and Control, with a total of 515 miRNAs in the collection of known/novel miRNAs that passed the filtering process of differential expression miRNA (DEGs) in either group. The 57 miRNAs were only uniquely found in the CPV, and 28 were only found in Control, with 430 being commonly expressed in both of the libraries (Fig. 4A).

To specify miRNAs in CPV infection, the normalized TPMs between the Control and CPV infected cells were wholly compared. The level of miRNA expression was differently changed. The abundance ranged from 0.1 TPM to 468,769 TPM in the Control and from 0.1 TPM to 474,115 TPM in the CPV group (GSE135948). After data normalization, eleven miRNAs were changed in abundance in CPV-infected cells by two-fold or more (log_2_ expression ratio ≥ 1 or ≤ 1). Among known differentially expressed miRNAs (DEGs), 3 were up-regulated (miR-222-5p, miR-365-2-5p, miR-1247-3p) and 2 (miR-322-5p, miR-361-3p) were down-regulated in the CPV significantly at the FDR < 0.05 level (Fig. 4B). The miR-17A-5p, miR-147-3p, miR-222-5p, miR-365-2-5p, miR-1247-3p, Novel 4 were upregulated miRNAs in CVP over Control libraries, and miR-322-5p, miR-361-3p, Novel 102, Novel 137, Novel 141 were downregulated miRNAs (Figs. 4C and 4D). In the case of novel miRNAs, the pattern was opposite, as three (Novel 102, Novel 137, Novel 141) were only down-regulated in the CPV group, no up-regulated novel miRNAs were found at the FDR < 0.05 (Fig. 4B). Of the 75 novel miRNAs that passed the filtering process, differentially expressed novel miRNAs demonstrated 2-fold or greater expression changes (log2 fold change; ≥ 1 or ≤ 1) between the Control and CPV groups were 22 (up-regulated) and 13 down-regulated novel miRNAs, respectively.

The DEGs with the DESeq2 package, we identified 6 upregulated and 5 downregulated genes using a threshold of FDR < 0.1 and fold-change > 2. The mean average (MA) plot is a plot for visual representation of genomic data. The plot visualizes the differences between measurements taken in two samples, by transforming the data onto log-fold change (M-values) and the MA (*A*-values) scales, then plotting these values. The MA plots are exploited to visualize high-throughput sequencing analysis. The MA and scatter plot of DEGs showed the predefined up-regulated genes; miR27A-5p, miR-147-3p, miR222-5p, miR365-2-5p and miR1247-3p, with one miRNA (novel 4) with never defined before (Fig. 4C). For those with downregulation in CPV-infected cells; miR361-5p, miR322-5p and 3 novel miRNAs (Novel 137, 141 and 102) were indicated with some novelty (FDR < 0.1).

**Figure 5:**
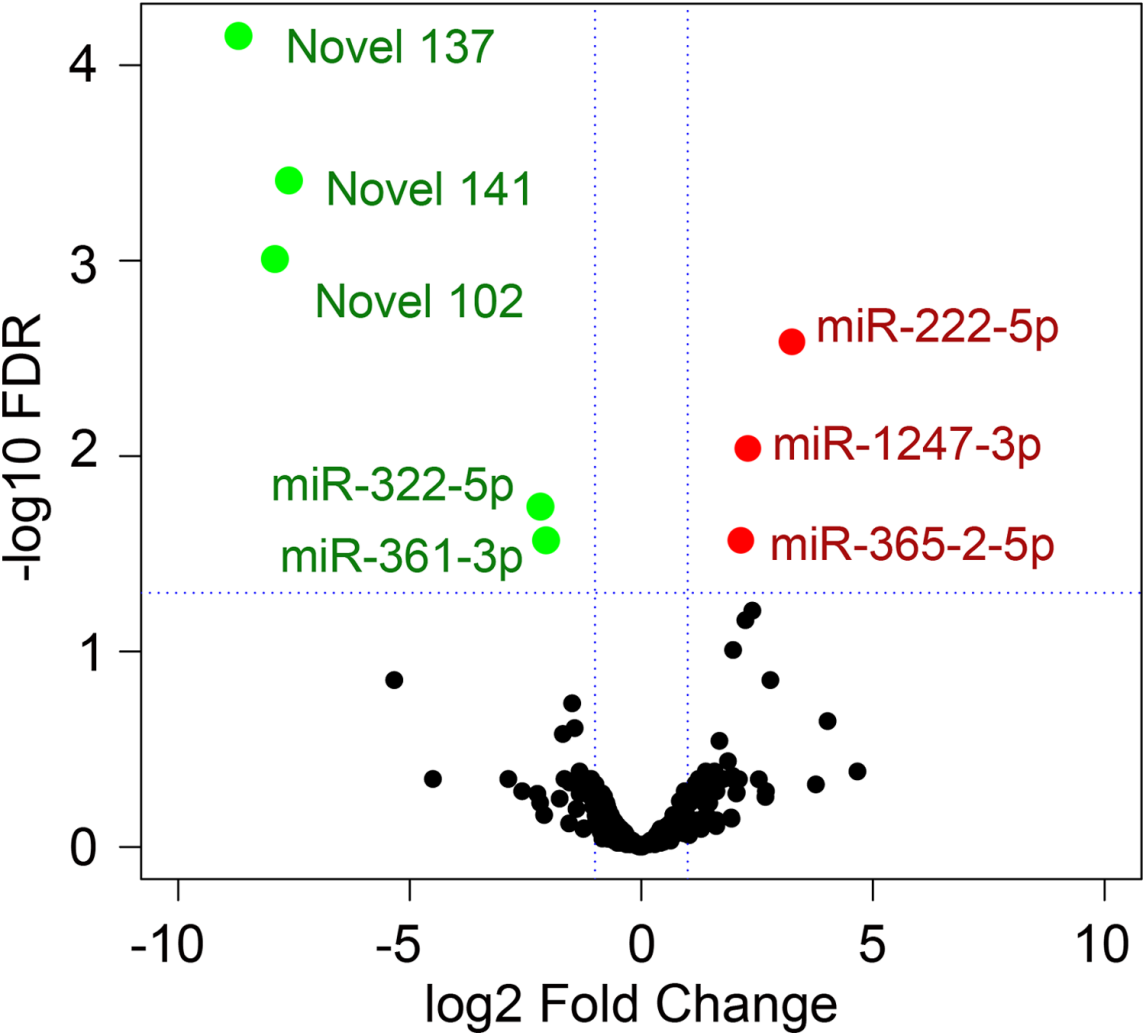
Volcano plot showed significant differentially expressed genes (DEGs). The *x*-axis shows the log2 fold change in gene expression, and the *y*-axis shows the statistical significance of the differences (FDR < 0.05, horizontal dotted line). Red dots indicate significant up-regulation, and green dots indicate down-regulation.

The Volcano plot could be used to infer the overall distribution of different expression miRNAs. For the experiment with biological replicate, as the DESeq2 has already eliminated the biological variation, our threshold was normally set as FDR < 0.05 (horizontal dotted line). Volcano plot of gene expression in CPV-infected cells showed fold change (vertical dotted lines) in gene expression regulated genes (FC ≥ 2 (red) or ≤ −2 (green), FDR < 0.05) are highlighted (Fig. 5). The volcano plot, the MA, and scatter plot show that CPV infection lead to a considerably minimal miRNA transcriptomic response. A quick scan at the top genes ranked by the absolute values of fold-change (FCs) told us that CPV induced miR-222-5p, miR-1247-3p and miR-365-2-5p whereas suppressed miR-361-3p, miR-322-5p, novel 102, 141 and 137 (based on FDR < 0.05).

### Prediction and annotation of potential target genes of differentially expressed miRNAs

**Figure 6:**
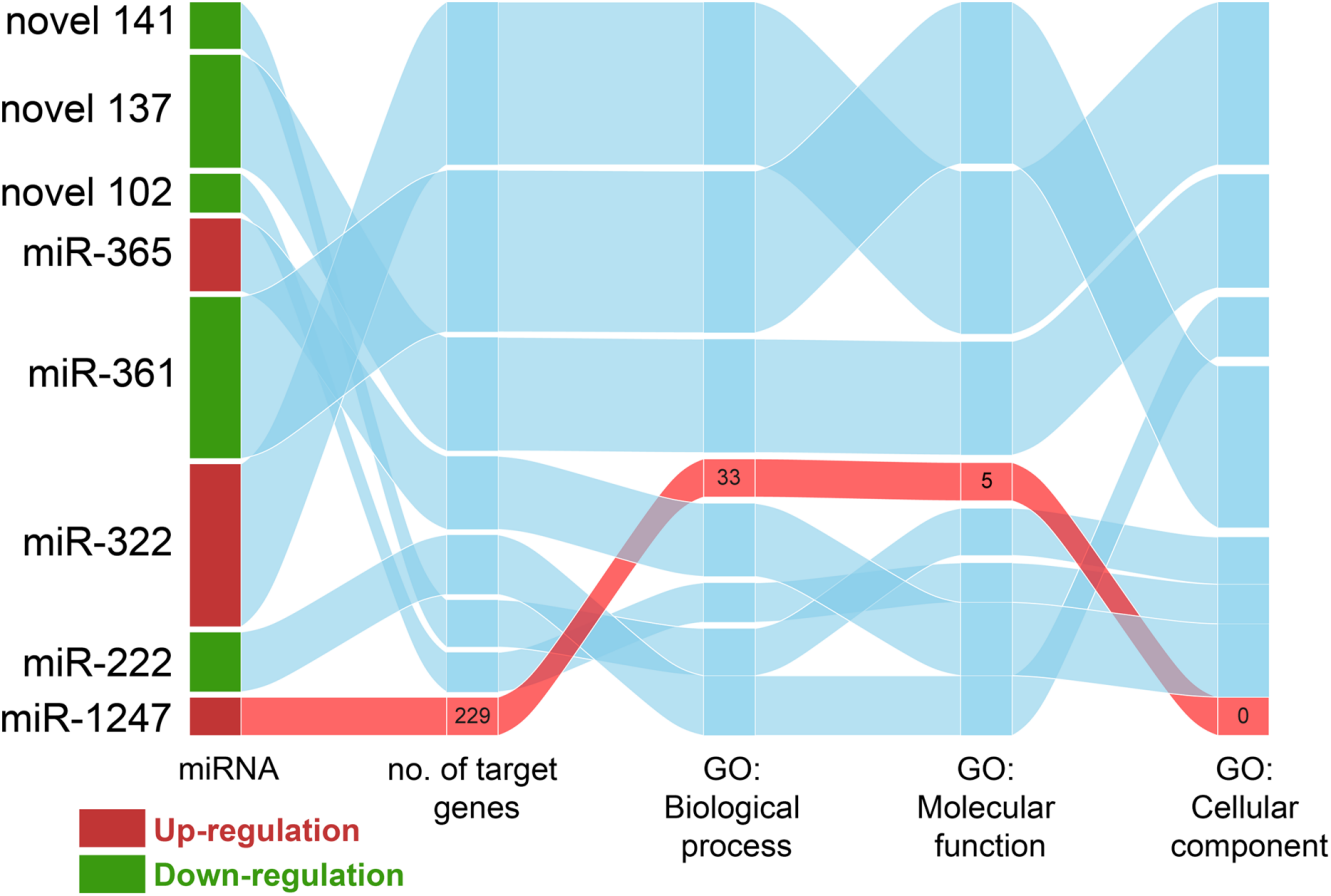
The relationship between miRNAs in CPV and target genes. The Sankey diagram shows significant miRNAs identified in CPV-infected libraries and numbers of their target genes, numbers of each related GO terms to given miRNAs. Only miRNA-1247 is highlighted in the diagram.

The sequence complementarity between miRNAs and target genes provides a quick and easy way to search for miRNAs target genes. A total of 5,828 target genes in *F. catus* genome was found in all 515 known and novel miRNAs identified in this study. A total of 3,182 target genes was identified in 5 out of the 8 significant differently expressed miRNAs (miR-222-5p, miR1247-3p, miR-365-2-5p, miR-322-5p and miR-361-3p). Predicting the target gene of known and novel miRNA have found the relationship between miRNA and target genes (Fig. 6; Table S7). The number of target genes for each differentially expressed miRNAs ranged from a few hundred to nearly a thousand. The highest number of target genes was predicted for miR-322-5p (972 genes), followed by miR-361-3p (965 genes), of which, both miRNAs were down-regulated in CPV infected cells (Table S8). However, the novel miRNAs; Novel 102, 137 and 141 also had several hundred target genes (Table S8).

**Figure 7:**
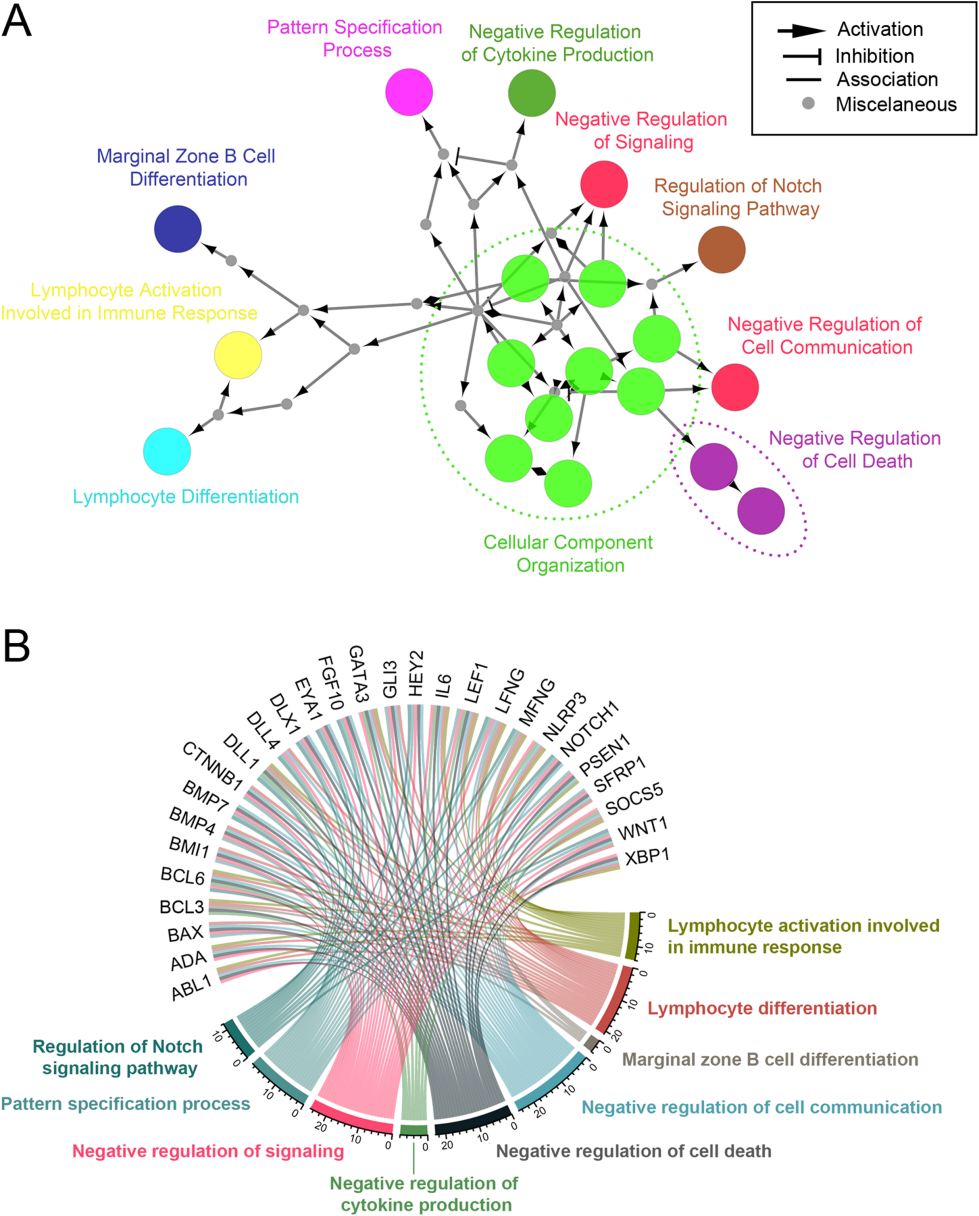
Enriched biological processes of miR-1247-3p gene targets. (A) ClueGO/CluePedia network shows a function group network with GO terms as nodes linked. The labels of the most significant term per group are shown. The node size depicts the significant enrichment. Functionally related groups (in part) are emphasized by overlapping of colors. (B) Chord diagram shows enriched biological processes (by colors), linking each gene cluster to the corresponding processes.

Of eight miRNA DEGs, we did test all up- and down-regulated miRNAs, but we chose only miR-1247-3p for further analysis. The predicted targets genes of up- and down-regulated miRNAs were then subjected to enrichment analysis based on the hypergeometric distribution. The GO Biological Process terms (BP) enriched in DEGs are shown in Fig. 7A and Table 1. Upregulated miRNA gene (miR-1247-3p) was related to regulation of cytokine and immune response, cell signaling, and cell death (FDR = 0.05). This is perhaps the cell’s response to infection of this particular virus by miRNA.

**Table 1:** Enrichment analysis of target genes of miR-1247-3p.

GO biological process	Gene (*n*)	Enrichment	FDR
Marginal zone B cell differentiation (GO:0002315)	3	50.12	2.82E−02
Mature B cell differentiation involved in immune response (GO:0002313)	3	37.59	3.93E−02
Phagocytosis, recognition (GO:0006910)	3	33.41	4.73E−02
Lymphocyte differentiation (GO:0030098)	9	6.68	1.38E−02
Lymphocyte activation (GO:0046649)	12	5.62	4.51E−03
T cell activation (GO:0042110)	8	5.61	3.64E−02
Leukocyte differentiation (GO:0002521)	10	5.25	1.81E−02
Leukocyte activation (GO:0045321)	12	4.63	1.39E−02
Pattern specification process (GO:0007389)	11	4.43	2.42E−02
Cell activation (GO:0001775)	13	4.19	1.60E−02
Immune system development (GO:0002520)	14	3.74	1.71E−02
Negative regulation of apoptotic process (GO:0043066)	16	3.43	1.74E−02
Negative regulation of programed cell death (GO:0043069)	16	3.36	1.71E−02
Negative regulation of cell death (GO:0060548)	16	3.08	2.99E−02
Negative regulation of signaling (GO:0023057)	21	2.94	1.27E−02
Negative regulation of signal transduction (GO:0009968)	19	2.81	2.51E−02
Negative regulation of cell communication (GO:0010648)	20	2.81	1.95E−02
Regulation of response to stimulus (GO:0048583)	46	2.01	7.87E−03
Negative regulation of cellular process (GO:0048523)	51	1.99	2.60E−03
Regulation of signal transduction (GO:0009966)	36	1.96	3.30E−02
Regulation of signaling (GO:0023051)	40	1.96	2.28E−02
Regulation of cell communication (GO:0010646)	38	1.87	3.99E−02
Cellular component organization (GO:0016043)	52	1.79	1.86E−02
Negative regulation of biological process (GO:0048519)	51	1.78	2.00E−02
Regulation of biological process (GO:0050789)	117	1.54	2.92E−05

**Figure 8:**
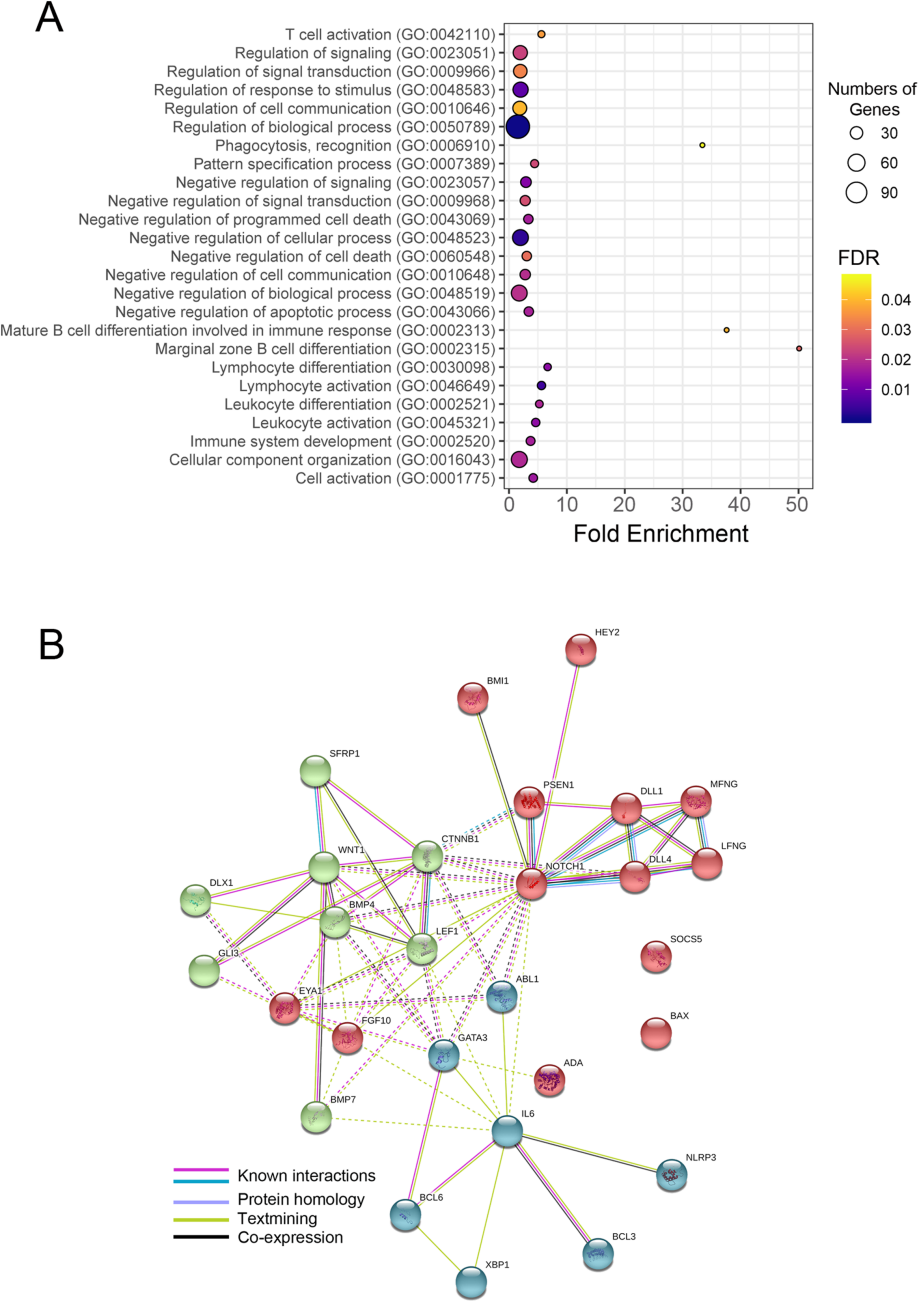
Gene ontology (GO) terms, numbers of the predicted target genes, and Protein-protein interaction network (PPI) of target genes of miR-1247-3p. (A) small RNA-seq data reveal GO biological processes among differential expressed genes in the CPV infected groups normalize with controls. The bubble plot represents the fold enrichment (*x*-axis), the number of genes (bubble size), and FDR value (gradient colors) in each GO biological process terms (*y*-axis). Representative bubbles were enriched at FDR < 0.05. (B) STRING protein–protein interaction network of co-expressed and interacted genes. The clusters of 28 genes in the network are grouped using color codes (red, blue and green).

Choosing GO biological process, we found that miRNA-1247-3p modulated at least 28 genes (Fig. 7B) that coded for proteins engaged in lymphocyte activation and differentiation, cytokine production, cell signaling and communication, antibody production, and cell death. As CPV infected stimulated the presence of miR-1247-3p and in turn, to regulate the production of many genes involved in the aforementioned processes, leading to reduction of proteins shared with the common pathways (Fig. 7B). GO categories were assigned for target genes of miR-1247-3p, and 229 putative targets were identified in the total of 38 enriched GO categories in terms of “biological process”, “cellular component” and “molecular function” (Fig. 6). Based on the biological process (BP), the genes were classified into 33 categories, of which the top ten over-represented GO terms (enrichment) were “lymphocyte activation involved in immune response”, “lymphocyte differentiation”, “marginal zone B cell differentiation”, “negative regulation of cytokine production”, “negative regulation of programed cell death”, “negative regulation of signaling”, “pattern specification process”, “regulation of Notch signaling pathway”, “response to organic substances” and “cellular component organization”, respectively (Fig. 8A).

The target genes were primarily varied with respect to function, as revealed by our analysis. In the case of molecular function, the genes were classified into five groups, of which they are mostly involved in “anion binding”, “protein binding” and “organic cyclic compound binding”. Based on cellular components, the genes were classified into zero categories. In biological process, the enrichments of genes were involved in “the immune system processes”, for example, “lymphocyte activation”, “phagocytosis recognition” and “leukocyte activation”. The most over-represented GO term (50.12-fold enrichment) was “marginal B cell zone differentiation”, involving three genes. Another significant in GO terms that could be emphasized here were “cell death process” and “cell signaling”, since those biological processes were found to be enriched in the target genes of miR-1247-3p. These two terms suggested that miRNAs are tightly involved in activation and signaling at the early phase of viral infection. At the advanced stage, the miRNAs are highly regulated with their targets in the process of the regulation of programed cell death, at the end (Fig. 8A; Table 1).

The other up- or down-regulated miRNAs in the current study possessed several hundred to many thousand target genes. No matter how many of the target genes it may involve, there were no significant biological processes that may have been highlighted the relevance in viral infection (Dataset S1). Of particular interest, novel miRNA, namely “Novel 137” may have been playing a pivotal role in the defense response to external stimuli. Since the “intracellular receptor signaling pathway” and “histone H4 acetylation” were two enrichment groups of genes that were highly correlated (Fig. S2). Furthermore, a gene network was constructed by utilizing the lists of genes involved in multiple biological process in order to illuminate the biological interpretation and meaningful. Of these target genes of differentially expressed miRNA, especially miR-1247-3p, a total of 28 genes was found to be highly diversified in multiple GO terms (Figs. 7B and 8B). In Figure 8B, the STRING network of PPIs among 28 diversified genes showed highly connected network included 26 genes at the core of the network. The 26 genes in the network were clustered into three groups, categorized by red, green and blue. Two genes (*BAX* and *SOCS5*) were excluded from the network, of which, were involved in apoptosis of the cells.

## Discussion

The outbreak and spread of the novel CPV-2 variant to Lao PDR (CPV-2c) have caused many significant health impacts on the dogs. The current dominated CPV Laotian strain (CPV-2c) has got the best fit in the animal resided in the urban environment, specifically, Vientiane capital of Laos (Vannamahaxay et al., 2017). To the best of our knowledge, CPV-2c is now reported globally as being more virulent than endemically strains. The alternation of amino acid at residue 426 (designated as CPv-2c) may play a critical role in pathogenicity of this virus. Based on the existing literatures and our additional findings, we speculated that the changes in miRNA gene expressions in the hosts infected with parvovirus family (e.g., CPV, MEV, FPV) would have the same but not identical pattern of miRNA expression (Sun et al., 2014; Zhou et al., 2017).

Introducing the next-generation sequencing technology, specifically, RNA sequencing (RNA-seq) has transformed the comprehensive measuring gene expression across the transcriptome, including small and miRNAs (Laganà et al., 2017; Sun et al., 2014; Zhou et al., 2017). This reliable platform with a high-throughput and powerful tool guarantees the enormous gene study. Across all RNA species, RNA-seq has revolutionized and become a widely used approach to study quantitative aspects of transcriptome profiling in the virus-infected cells (Li et al., 2019; Sun et al., 2014, 2019; Zhou et al., 2017). In the current study, we have explored the miRNA milieu in CRFK cells infected with CPV-2c Laotian isolates (Puentes et al., 2012). We have identified 515 DEGs using sRNA libraries in either uninfected control or CPV-infection. In the literatures, CRFK cell line has been extensively characterized for its use as a standard model system to investigate CPV infection (Raj et al., 2010; Yi et al., 2016; Yoshikawa et al., 2010), but miRNA sequencing platform in CRFK cells had not been previously reported or studied.

In genomics era, we have a chance to execute our aims to analyze large datasets from CRFK samples from CPV individuals using sRNA-seq data to enumerate, quantify, assess and comprehend the heterogeneity of DEGs during in vitro experimental CPV infection. Compared to recent evidence reported by Sun and coworkers (Sun et al., 2014), they extensively analyzed the DEGs from miRNA-seq data of the MEV in feline kidney (F81) cell line. They have identified 196 known mammalian miRNAs in their report. Similar to our findings, we have found more known miRNAs in CPV-infected CRFK cells (420 of known miRNAs). Additionally, in MEV-infected cells, they found a minimal number of miRNAs that significantly down-regulated (miR-181b, miR-181d and miR-301) and up-regulated (miR-29b, miR-125b and miR-320a). Consistent with our results, we pinpointed of about three miRNAs that significantly up-regulated in CPV infection (miR-222-5p, miR-1247-3p and miR-365-5p) whereas many more miRNAs were significantly down-regulated (miR-322-5p and miR-361-3p) including Novel 137, Novel 141 and Novel 102 miRNAs. The differences in miRNA species found by two studies reflect the effects of various factors, for example, the virus and cell line used in the studies, which makes it very difficult to compare the results side by side.

Our current CPV bioinformatic aspects revealed the correlation of CPV miRNA-seq data has a larger dynamic range of its target genes. Within five preferential miRNAs with significant DEGs (miR-222-5p, miR1247-3p, miR-365-2-5p, miR-322-5p and miR-361-3p), we have identified more than 3,000 target genes. In CRFK infected cells, the gene targets of significant miRNA DEGs functionally belong to immune response (e.g., lymphocyte activation and antibody production), cell communication and signaling, cytokine production, and lastly cell death. Consistent with our results, a similar relevance of gene network in the signaling pathways of virus-host interaction was reported by Fan et al. (2016) in CPV-2a infected MDCK cells. In Zhou et al. (2017), the genes pertaining metabolism-related pathways, cytokine- and pathogen-host interaction-related pathway were affected in feline parvovirus (FPV) infected cats.

Our initial screening of DEGs defined the three most significant upregulated miRNA genes were miR-222-5p, miR-1247-3p and miR-365-2-5p. Of particular interests in immunological response to the virus, miR-1247-3p had roughly 229 target genes, whose function can be categorized into 33 GO by GO enrichment analysis (GO biological process term). The miRNA-1247-3p function is estimated to modulate at least 200 genes, involving in 38 GO biological processes (BP) plus molecular function (MF) due to the redundancy with partial target recognition. As stated earlier, miRNA-1247-3p could, at least, be regulated 28 functional genes in our reports, but the actual numbers would be much higher. Our findings in CPV-infection reveal an exceptional function of miR-1247-3p in response to virus, other than the functions as onco-miRNA and controlling of cartilage function (Fang et al., 2018; Martinez-Sanchez & Murphy, 2013).

The functional genes in the GO process can be overlapping at certain points in the pathway, though this was obviously modulated by miR-1247-3p in this study. For example, we found that genes: *ABL1*, *ADA, BAX, BCL3, BCL6, BMI1, BMP4, CTNNB1, DLL1, GATA3, GLI3, IL6, LEF1, NLRP3, PSEN1, SFRP1, SOCS5, WNT1, XBP1* can be partially intersected in lymphocyte activation involved in immune response, lymphocyte differentiation, negative regulation of cell communication, and negative regulation of cell death pathways. When many affected genes by miRNAs have converged, it will be amplified the signals or expansion of its functions to adjacent gene network. The synergism among genes within GO biological processes in reacting to virus infections, as anticipated in CPV as well, is a common paradigm for effective host defense response. Of the thirty-three GO process categories discovered in the current study, IL-6 was remarkably important cytokine, as a key for marginal zone B cell differentiation, negative regulation of cytokine production, and lymphocyte differentiation, by its action (Safi et al., 2017; Zhou et al., 2018).

This work has highlighted the importance of some potential miRNAs and their target functional, relevant genes in controlling and mitigating immune response to CPV virus. Whilst most analyzed miRNAs in our findings remained unchanged (430 miRNAs) due to their minimal roles in response to virus, some are apparently shedding light on the role of the manipulating of several GO pathways. The miR-1247-3p, accompanying by other significant DEGs, as earlier discussed, can be linked to further experimental observations, leading directions for further work and implication for clinical use.

## Conclusions

We have identified the DEGs in the miRNA repertoire during CPV-2c infection of CRFK cells. Our work revealed that the miRNA-1247-3p and its cognate target genes in biological pathways highlight their great potential as novel therapeutic targets or diagnostic biomarkers of CPV infection.

## Supplemental Information

10.7717/peerj.8522/supp-1 Supplemental Information 1 Statistical summary of the data that were generated by high-throughput small-RNA sequencing in *F. catus* microRNAs.

10.7717/peerj.8522/supp-2 Supplemental Information 2 Small RNA annotation.

10.7717/peerj.8522/supp-3 Supplemental Information 3 Statistics of mapping results.

10.7717/peerj.8522/supp-4 Supplemental Information 4 Summary of known miRNA in each sample.

10.7717/peerj.8522/supp-5 Supplemental Information 5 Summary of novel miRNA.

10.7717/peerj.8522/supp-6 Supplemental Information 6 List of miRNA family identified in the current study.

10.7717/peerj.8522/supp-7 Supplemental Information 7 Prediction of target genes of known and novel miRNAs (significant differently expressed miRNAs) in the current study.

10.7717/peerj.8522/supp-8 Supplemental Information 8 List of significant differently expressed miRNAs, prediction of number of target genes, and number of related gene ontology (GO) functional analysis identified in the current study.

10.7717/peerj.8522/supp-9 Supplemental Information 9 First nucleotide bias and nucleotide bias at each position of novel feline miRNAs. (A) First nucleotide bias of novel *F. catus* miRNA candidates in Control and CPV libraries. The number on top of the bars indicated the number of sequences corresponding to the miRNA length (nt). *x*-axis, length of miRNAs; *y*-axis, responding percent of each nucleotide. (B) Nucleotide bias at each position in Control and CPV libraries. *x*-axis, Position of nucleotide in miRNAs; *y*-axis, responding percent of each nucleotide.

10.7717/peerj.8522/supp-10 Supplemental Information 10 Enriched biological processes of Novel 137 miRNA gene targets. ClueGO/CluePedia network shows a function group network with GO terms as nodes linked of target genes of Novel 137. The labels of the most significant term per group are shown. The node size depicts the significant enrichment. Functionally related groups (in part) are emphasized by overlapping of colors.

10.7717/peerj.8522/supp-11 Supplemental Information 11 Raw data of Gene Ontology (GO) enrichment analysis. GO enrichment analysis of target genes of differentially expressed miRNA genes (miR-222, miR-322, miR-361, miR-365, miR-1247 and Novel-137) by g:Profiler and PANTHER GO Enrichment Analysis.

## Additional Information and Declarations

### Data Availability

The datasets or raw files of sRNA-seq generated during and/or analyzed during the current study are available through the Gene Expression Omnibus (GEO: GSE135948) and in the Supplemental Tables.
