# Supplementary material for: Detection and characterization of microRNA expression profiling and its target genes in response to canine parvovirus in Crandell Reese Feline Kidney cells
Source: PeerJ. 2020 Feb 12;8:e8522. doi: 10.7717/peerj.8522 (PMC7023829; doi:10.7717/peerj.8522)
Supplement: Supplemental Information 1 [file peerj-08-8522-s001.docx]

**Supplementary Table 1** **Statistical summary of the data that were generated by high-throughput small-RNA sequencing in *Felis catus* microRNAs.**

| Sample | Total raw reads | High quality reads | Low quality reads | N% > 10% | 5' adapter contaminants | 3' adapter null | Poly  A/T/G/C |
| --- | --- | --- | --- | --- | --- | --- | --- |
| Control 01 | 25,230,038  (100.00%) | 24,891,422  (98.66%) | 100,575  (0.40%) | 0  (0.00%) | 3,776  (0.01%) | 181,678 (0.72%) | 52,587  (0.21%) |
| Control 02 | 25,410,594  (100.00%) | 24,923,852  (98.08%) | 100,174  (0.39%) | 0  (0.00%) | 4,707  (0.02%) | 298,854 (1.18%) | 83,007  (0.33%) |
| CPV 01 | 23,466,804  (100.00%) | 22,909,717  (97.63%) | 81,129  (0.35%) | 0  (0.00%) | 3,228  (0.01%) | 431,286 (1.84%) | 41,444  (0.18%) |
| CPV 02 | 26,337,331  (100.00%) | 25,935,795  (98.48%) | 113,187  (0.43%) | 53  (0.00%) | 3,876  (0.01%) | 253,129 (0.96%) | 31,291  (0.12%) |

N%, undetectable gaps at base calling
