# Supplementary material for: Detection and characterization of microRNA expression profiling and its target genes in response to canine parvovirus in Crandell Reese Feline Kidney cells
Source: PeerJ. 2020 Feb 12;8:e8522. doi: 10.7717/peerj.8522 (PMC7023829; doi:10.7717/peerj.8522)
Supplement: Supplemental Information 2 [file peerj-08-8522-s002.docx]

**Supplementary Table 2 Small RNA annotation.**

| **Types** | **Control 01** | **Control 01**  **(percent)** | **Control 02** | **Control 02**  **(percent)** | **CPV 01** | **CPV 01**  **(percent)** | **CPV 02** | **CPV02**  **(percent)** |
| --- | --- | --- | --- | --- | --- | --- | --- | --- |
| Total | 17,492,270 | 100.00% | 17,464,428 | 100.00% | 15,118,021 | 100.00% | 19,220,070 | 100.00% |
| Known miRNA | 11,065,272 | 63.26% | 9,059,190 | 51.87% | 11,148,071 | 73.74% | 15,632,155 | 81.33% |
| rRNA | 1,366,005 | 7.81% | 2,303,723 | 13.19% | 171,338 | 1.13% | 127,277 | 0.66% |
| tRNA | 1,098,446 | 6.28% | 1,474,379 | 8.44% | 5,769 | 0.04% | 11,811 | 0.06% |
| snRNA | 14,687 | 0.08% | 8,997 | 0.05% | 32,343 | 0.21% | 24,545 | 0.13% |
| snoRNA | 313,929 | 1.79% | 351,827 | 2.01% | 575,467 | 3.81% | 489,399 | 2.55% |
| Repeat | 542,318 | 3.10% | 434,031 | 2.49% | 739,481 | 4.89% | 964,320 | 5.02% |
| Novel miRNA | 4,642 | 0.03% | 5,313 | 0.03% | 8,718 | 0.06% | 6,582 | 0.03% |
| Exon: + | 1,615,593 | 9.24% | 1,930,699 | 11.06% | 507,223 | 3.36% | 581,837 | 3.03% |
| Exon: − | 251,605 | 1.44% | 353,900 | 2.03% | 15,620 | 0.10% | 10,806 | 0.06% |
| Intron: + | 239,028 | 1.37% | 278,728 | 1.60% | 514,908 | 3.41% | 442,197 | 2.30% |
| Intron: − | 101,556 | 0.58% | 136,432 | 0.78% | 86,840 | 0.57% | 62,403 | 0.32% |
| Other | 879,189 | 5.03% | 1,127,209 | 6.45% | 1,312,243 | 8.68% | 866,738 | 4.51% |
