# Supplementary material for: Detection and characterization of microRNA expression profiling and its target genes in response to canine parvovirus in Crandell Reese Feline Kidney cells
Source: PeerJ. 2020 Feb 12;8:e8522. doi: 10.7717/peerj.8522 (PMC7023829; doi:10.7717/peerj.8522)
Supplement: Supplemental Information 3 [file peerj-08-8522-s003.docx]

**Supplementary Table 3** **Statistics of mapping results.**

| **Sample** | **Total sRNA** | **Mapped sRNA** | **"+" Mapped sRNA** | **"−" Mapped sRNA** |
| --- | --- | --- | --- | --- |
| **Control 01** | 22,740,485 (100.00%) | 17,492,270 (76.92%) | 8,334,604 (36.65%) | 9,157,666 (40.27%) |
| **Control 02** | 22,252,712 (100.00%) | 17,464,428 (78.48%) | 9,173,199 (41.22%) | 8,291,229 (37.26%) |
| **CPV 01** | 21,476,489 (100.00%) | 15,118,021 (70.39%) | 5,023,904 (23.39%) | 10,094,117 (47.00%) |
| **CPV 02** | 25,046,386 (100.00%) | 19,220,070 (76.74%) | 6,811,975 (27.20%) | 12,408,095 (49.54%) |

"+" Mapped sRNA: Quantity and percentage of mapped sRNAs in the same direction as the genome

"-" Mapped sRNA: Quantity and percentage of mapped sRNAs in the opposite direction as the genome
