# Supplementary material for: Detection and characterization of microRNA expression profiling and its target genes in response to canine parvovirus in Crandell Reese Feline Kidney cells
Source: PeerJ. 2020 Feb 12;8:e8522. doi: 10.7717/peerj.8522 (PMC7023829; doi:10.7717/peerj.8522)
Supplement: Supplemental Information 4 [file peerj-08-8522-s004.docx]

**Supplementary Table 4** **Summary of known miRNA in each sample.**

| **Types** | **Total** | **Control 01** | **Control 02** | **CPV 01** | **CPV 02** |
| --- | --- | --- | --- | --- | --- |
| Mapped mature | 420 | 373 | 377 | 375 | 395 |
| Mapped hairpin | 333 | 293 | 297 | 297 | 301 |
| Mapped unique sRNA | 14,839 | 3,622 | 3,466 | 3,665 | 4,086 |
| Mapped total sRNA | 46,904,688 | 11,065,272 | 9,059,190 | 11,148,071 | 15,632,155 |
