# Supplementary material for: Detection and characterization of microRNA expression profiling and its target genes in response to canine parvovirus in Crandell Reese Feline Kidney cells
Source: PeerJ. 2020 Feb 12;8:e8522. doi: 10.7717/peerj.8522 (PMC7023829; doi:10.7717/peerj.8522)
Supplement: Supplemental Information 5 [file peerj-08-8522-s005.docx]

**Supplementary Table 5 Summary of novel miRNA.**

| **Types** | **Total** | **Control 01** | **Control 02** | **CPV 01** | **CPV 02** |
| --- | --- | --- | --- | --- | --- |
| Mapped mature | 140 | 100 | 101 | 120 | 115 |
| Mapped star | 68 | 31 | 36 | 35 | 42 |
| Mapped hairpin | 141 | 107 | 110 | 124 | 121 |
| Mapped unique sRNA | 1,356 | 294 | 307 | 366 | 389 |
| Mapped total sRNA | 25,255 | 4,642 | 5,313 | 8,718 | 6,582 |
