# Supplementary material for: Detection and characterization of microRNA expression profiling and its target genes in response to canine parvovirus in Crandell Reese Feline Kidney cells
Source: PeerJ. 2020 Feb 12;8:e8522. doi: 10.7717/peerj.8522 (PMC7023829; doi:10.7717/peerj.8522)
Supplement: Supplemental Information 6 [file peerj-08-8522-s006.docx]

**Supplementary Table 6 List of miRNA family identified in the current study.**

| let-7 | mir-32 | mir-139 | mir-188 | mir-297 | mir-362 | mir-467 | mir-653 | mir-1839 |
| --- | --- | --- | --- | --- | --- | --- | --- | --- |
| mir-1 | mir-33 | mir-140 | mir-190 | mir-299 | mir-363 | mir-483 | mir-654 | mir-1843 |
| mir-7 | mir-34 | mir-142 | mir-191 | mir-302 | mir-365 | mir-484 | mir-670 | mir-1892 |
| mir-8 | mir-95 | mir-143 | mir-192 | mir-320 | mir-367 | mir-485 | mir-671 | mir-1893 |
| mir-9 | mir-96 | mir-144 | mir-193 | mir-322 | mir-368 | mir-486 | mir-675 | mir-1898 |
| mir-10 | mir-101 | mir-145 | mir-194 | mir-324 | mir-370 | mir-490 | mir-680 | mir-1902 |
| mir-15 | mir-103 | mir-146 | mir-196 | mir-325 | mir-374 | mir-491 | mir-684 | mir-1949 |
| mir-17 | mir-122 | mir-147 | mir-199 | mir-326 | mir-375 | mir-493 | mir-703 | mir-1954 |
| mir-19 | mir-124 | mir-148 | mir-202 | mir-328 | mir-378 | mir-497 | mir-708 | mir-3059 |
| mir-21 | mir-126 | mir-149 | mir-203 | mir-329 | mir-379 | mir-499 | mir-744 | mir-3064 |
| mir-22 | mir-127 | mir-150 | mir-204 | mir-330 | mir-423 | mir-500 | mir-760 | mir-3065 |
| mir-23 | mir-128 | mir-153 | mir-205 | mir-331 | mir-425 | mir-503 | mir-762 | mir-3074 |
| mir-24 | mir-129 | mir-154 | mir-210 | mir-335 | mir-433 | mir-504 | mir-873 | mir-3085 |
| mir-25 | mir-130 | mir-181 | mir-214 | mir-338 | mir-448 | mir-505 | mir-874 | mir-3154 |
| mir-26 | mir-132 | mir-182 | mir-216 | mir-339 | mir-449 | mir-542 | mir-877 | mir-3535 |
| mir-27 | mir-133 | mir-183 | mir-218 | mir-340 | mir-450 | mir-574 | mir-881 | mir-3618 |
| mir-28 | mir-134 | mir-184 | mir-219 | mir-342 | mir-451 | mir-590 | mir-935 | mir-6516 |
| mir-29 | mir-135 | mir-185 | mir-221 | mir-345 | mir-452 | mir-592 | mir-1247 |  |
| mir-30 | mir-137 | mir-186 | mir-223 | mir-346 | mir-455 | mir-615 | mir-1298 |  |
| mir-31 | mir-138 | mir-187 | mir-290 | mir-361 | mir-466 | mir-652 | mir-1306 |  |
