# Supplementary material for: Detection and characterization of microRNA expression profiling and its target genes in response to canine parvovirus in Crandell Reese Feline Kidney cells
Source: PeerJ. 2020 Feb 12;8:e8522. doi: 10.7717/peerj.8522 (PMC7023829; doi:10.7717/peerj.8522)
Supplement: Supplemental Information 7 [file peerj-08-8522-s007.docx]

**Supplementary Table 7**

**Prediction of target genes of known and novel miRNAs (significant differently expressed miRNAs) in the current study.** Abbreviated gene symbols are shown.

| miR-361-3p | miR-322-5p | miR-365-2-5p | miR-1247-3p | miR-222-5p | Novel 137 | Novel 141 | Novel 102 |
| --- | --- | --- | --- | --- | --- | --- | --- |
| *A2ML1* | *AAMP* | *AADACL3* | *ABCC1* | *AADACL3* | *AASS* | *AAMP* | *AASDH* |
| *AAMP* | *AATF* | *AATF* | *ABHD11* | *ABCA5* | *ABHD11* | *AASDH* | *ABCA5* |
| *ABCA3* | *ABCC1* | *ABCF1* | *ABLIM2* | *ABCB9* | *ABT1* | *AASS* | *ADAMTS4* |
| *ABCB9* | *ABCC5* | *ABHD8* | *ACTR10* | *ABCC1* | *ACHE* | *ABHD11* | *ADHFE1* |
| *ABCC5* | *ABI2* | *ABLIM3* | *ADAMTS18* | *ACADM* | *ACOT11* | *ACKR2* | *ADIG* |
| *ABHD11* | *ACCS* | *ACTB* | *ADSS* | *ADAMTS4* | *ADAM23* | *ACVRL1* | *AEBP2* |
| *ABI3* | *ACKR2* | *ACVRL1* | *AFAP1* | *ADCYAP1* | *ADAMTSL1* | *ADH6* | *AGTPBP1* |
| *ABLIM3* | *ACSS2* | *ADAM23* | *AFG3L2* | *AFAP1L1* | *ADAMTSL4* | *AKAP6* | *ANKRD29* |
| *ACOT11* | *ACTG1* | *ADAMTS14* | *AKIRIN1* | *AHSA1* | *ADORA2A* | *ALDH1L1* | *AP3S1* |
| *ACTRT3* | *ACTR10* | *ADAMTS18* | *ALDH1A2* | *AJAP1* | *AFAP1L1* | *ANO10* | *APPL1* |
| *ADAMTS12* | *ACVR2A* | *ADCY5* | *AMN* | *AKAIN1* | *AFG3L2* | *APBB2* | *ARHGAP5* |
| *ADAMTS14* | *ADAMTS18* | *ADSS* | *ANKRD40* | *AKIRIN1* | *AGBL2* | *ARHGAP5* | *ARHGEF26* |
| *ADAMTS19* | *ADCY5* | *AFAP1* | *AREL1* | *ALDH1A2* | *ALDH18A1* | *ARHGAP6* | *ARMC10* |
| *ADAP2* | *ADGRA2* | *AFAP1L1* | *ARHGAP32* | *ALDH3A2* | *ALDH1A2* | *ARHGEF26* | *ATP2B3* |
| *ADCY5* | *ADGRB2* | *AJAP1* | *ARHGAP35* | *ALPK1* | *ALDH1L1* | *ARID4A* | *B4GALT6* |
| *ADCY7* | *ADGRD1* | *AKAP1* | *ARHGAP6* | *AMPD3* | *ALDH3A1* | *ARRB1* | *BCL2L15* |
| *ADGRA2* | *ADGRL2* | *ALDH3A1* | *ARHGEF10* | *AP1S3* | *ALX1* | *ARRDC3* | *BMP5* |
| *ADGRB2* | *ADNP2* | *ANGPTL7* | *ARRB1* | *APEX1* | *ANAPC5* | *AVPR1A* | *C11orf97* |
| *ADGRD1* | *ADORA2A* | *ANKFN1* | *ATP2B3* | *APLP2* | *ANO10* | *BABAM2* | *C4orf33* |
| *ADGRG5* | *ADRA2B* | *ANKRD40* | *BCL6* | *AREL1* | *ANXA6* | *BAG2* | *C4orf54* |
| *ADIG* | *AFAP1* | *ANTXR2* | *BCL7A* | *ARHGAP18* | *AP1B1* | *BCAM* | *C8orf88* |
| *ADIPOR2* | *AGBL2* | *ANXA13* | *BCOR* | *ARIH2* | *AP1G1* | *BCL11A* | *CACNB2* |
| *ADORA2A* | *AHSA1* | *AP1G1* | *C19orf25* | *ARMC2* | *AP1M2* | *BMP5* | *CAP2* |
| *ADPRHL2* | *AIP* | *ARHGAP35* | *CACNA1I* | *ARMC8* | *AP3B1* | *BRWD3* | *CASKIN2* |
| *ADRA2B* | *AJAP1* | *ARHGAP5* | *CAV3* | *ATE1* | *APBA1* | *BUB1* | *CAV2* |
| *AFAP1* | *AKAP1* | *ARNTL* | *CCDC151* | *ATG5* | *APEX1* | *C12orf4* | *CBLL1* |
| *AGFG1* | *AKAP11* | *ARPC2* | *CCER2* | *ATP13A5* | *ARHGAP35* | *C14orf28* | *CBX2* |
| *AHSA1* | *AKAP7* | *ARRB1* | *CCNG2* | *AVPR1A* | *ARHGEF10* | *C18orf54* | *CCDC82* |
| *AJAP1* | *AKIP1* | *ARRDC1* | *CCT5* | *BAAT* | *ARHGEF15* | *C20orf194* | *CD2AP* |
| *AKAP1* | *AKT3* | *ASPN* | *CCT7* | *BICRAL* | *ARHGEF16* | *C4orf54* | *CD53* |
| *AKAP7* | *ALAD* | *ATP6V0D2* | *CD44* | *BMS1* | *ARID3C* | *C5orf24* | *CD9* |
| *ALDH1L1* | *ALDH1A2* | *BABAM2* | *CD81* | *C19orf25* | *ARIH2* | *C5orf51* | *CDK12* |
| miR-361-3p | **miR-322-5p** | **miR-365-2-5p** | **miR-1247-3p** | **miR-222-5p** | **Novel 137** | **Novel 141** | **Novel 102** |
| *ALOX5* | *AMFR* | *BARHL2* | *CDH8* | *C4orf33* | *ARL5C* | *CA3* | *CDKN2AIPNL* |
| *ALPK1* | *ANGEL1* | *BCL2L15* | *CDK18* | *C4orf54* | *ARMC10* | *CACNA1H* | *CELF1* |
| *ANAPC5* | *ANK3* | *BCLAF3* | *CDK9* | *CASKIN2* | *ARRB1* | *CALHM5* | *CELF5* |
| *ANGPTL4* | *ANKFN1* | *BHMT2* | *CEP85* | *CASP14* | *ASCC3* | *CAPZA2* | *CHFR* |
| *ANGPTL7* | *ANKIB1* | *BRD9* | *CHD4* | *CAT* | *ATG4D* | *CBLN1* | *CHRNA1* |
| *ANO10* | *ANKRD13D* | *C11orf49* | *CHD6* | *CBFA2T2* | *ATP6V1F* | *CCDC88C* | *CHRNA3* |
| *ANXA13* | *ANKRD29* | *C17orf75* | *CIT* | *CBLL1* | *AUNIP* | *CCNG2* | *CHRNB3* |
| *AP1G1* | *ANO6* | *C1QL1* | *CKAP4* | *CBR3* | *BAG6* | *CCR7* | *CLEC5A* |
| *AP2A1* | *ANXA3* | *C2orf68* | *CLCF1* | *CC2D2A* | *BAIAP2L1* | *CD53* | *CNTN3* |
| *AP3D1* | *AP1B1* | *C8orf88* | *CLEC7A* | *CCDC150* | *BCAS4* | *CDCP1* | *COL3A1* |
| *AP4M1* | *AP1G1* | *CA3* | *CLUH* | *CCDC85A* | *BCL11A* | *CDK15* | *COPS3* |
| *APBA1* | *AP1S3* | *CACNB3* | *CMTM3* | *CCDC88C* | *BCLAF3* | *CEP162* | *CPXM2* |
| *APEX1* | *AP2A1* | *CALB2* | *CNOT9* | *CCNJL* | *BET1L* | *CHAT* | *CTBS* |
| *APLP2* | *APBA1* | *CARNMT1* | *CNRIP1* | *CCR7* | *BHMT2* | *CHD5* | *CTNNBIP1* |
| *APPBP2* | *APP* | *CBFA2T2* | *COQ8A* | *CCT7* | *BPIFB2* | *CHEK1* | *CYP39A1* |
| *AQP4* | *APPBP2* | *CCDC137* | *CREB3L1* | *CD2AP* | *BPIFB3* | *CHMP7* | *DAB1* |
| *ARFGEF2* | *AQP4* | *CCDC25* | *CRHR2* | *CD44* | *BRD9* | *CHRNB3* | *DCAF7* |
| *ARFRP1* | *AREL1* | *CCDC32* | *CTBP1* | *CDC40* | *BRINP2* | *CIT* | *DCK* |
| *ARHGAP35* | *ARHGAP12* | *CCKBR* | *CTNNA1* | *CELF1* | *BRPF3* | *CLSTN2* | *DCLK1* |
| *ARHGAP44* | *ARHGAP23* | *CCND2* | *CTNNBL1* | *CELF2* | *C11orf49* | *CNNM4* | *DCP1A* |
| *ARHGEF16* | *ARHGAP32* | *CCNY* | *CUL4A* | *CELSR2* | *C16orf70* | *CNOT9* | *DCT* |
| *ARHGEF17* | *ARHGAP35* | *CD22* | *CYB5R1* | *CEP85L* | *C17orf75* | *CNTN4* | *DISP3* |
| *ARHGEF7* | *ARHGAP5* | *CD44* | *CYP26B1* | *CES1* | *C18orf21* | *CRISPLD1* | *DNAJC19* |
| *ARID3A* | *ARHGAP6* | *CDC42BPB* | *DDX59* | *CFL2* | *C19orf25* | *CTIF* | *DNAJC25* |
| *ARID5A* | *ARHGEF10* | *CDK15* | *DENND2A* | *CHAD* | *C19orf84* | *CTNND2* | *EBAG9* |
| *ARL10* | *ARHGEF17* | *CDK18* | *DLL1* | *CHCHD6* | *C1orf109* | *CWC22* | *EIF5* |
| *ARL14EP* | *ARHGEF7* | *CDKN2A* | *DNAJC16* | *CHD1L* | *C1orf115* | *CXCL10* | *EIF5A2* |
| *ARMH3* | *ARID5A* | *CEP162* | *DNAJC5* | *CHD4* | *C1orf158* | *CYBRD1* | *ELF5* |
| *ARPC1B* | *ARL10* | *CFAP161* | *DOLPP1* | *CHP2* | *C1QA* | *CYP2R1* | *ELOVL6* |
| *ARPC2* | *ARL14EP* | *CHRNA4* | *DPP6* | *CHST10* | *C1QC* | *DAPK2* | *ELP2* |
| *ARRB1* | *ARMC8* | *CHRNA6* | *DRAXIN* | *CHST3* | *C1QL1* | *DCP2* | *ETF1* |
| *ARRDC1* | *ARMH3* | *CHST10* | *DRP2* | *CLTCL1* | *C1QTNF7* | *DDRGK1* | *EXOC2* |
| *ATF5* | *ARRB1* | *CLCN4* | *EHMT1* | *CNOT11* | *C5orf15* | *DELE1* | *FAM104A* |
| *ATG13* | *ARRDC1* | *CNGB3* | *ELAVL1* | *COL11A1* | *CACFD1* | *DENND2A* | *FBXL18* |
| *ATG4D* | *ATF3* | *COG5* | *ELF4* | *COPS7A* | *CACHD1* | *DHRS1* | *FBXO28* |
| *ATP13A3* | *ATG13* | *COL11A1* | *EML4* | *CPD* | *CACNA1H* | *DLG3* | *FBXO45* |
| *ATP1B1* | *ATG14* | *COL3A1* | *EPB41* | *CSGALNACT1* | *CACNA1I* | *DNAJC3* | *FGB* |
| *ATP6V1G1* | *ATG5* | *COL9A2* | *EPB41L1* | *CTDP1* | *CACNB3* | *DPY19L3* | *FIP1L1* |
| miR-361-3p | **miR-322-5p** | **miR-365-2-5p** | **miR-1247-3p** | **miR-222-5p** | **Novel 137** | **Novel 141** | **Novel 102** |
| *ATP8A2* | *ATP13A3* | *COPS7A* | *EPHX1* | *CTSC* | *CACNG4* | *DUSP1* | *FMNL3* |
| *BABAM2* | *ATP2B3* | *CRLF3* | *ETV3* | *CTSE* | *CANX* | *ECH1* | *FNBP1L* |
| *BAG6* | *ATP6V0A2* | *CTDSP1* | *ETV4* | *CXCL17* | *CAPN5* | *EHD4* | *FNIP1* |
| *BAHD1* | *ATP6V1A* | *CTNNBL1* | *FADS2* | *DAZAP1* | *CARM1* | *ELP2* | *FOXO3* |
| *BAIAP2* | *ATP6V1C1* | *CYP26B1* | *FBXO44* | *DCDC2* | *CARNS1* | *EML4* | *FUBP3* |
| *BAZ1B* | *BABAM2* | *DAAM2* | *FGFR1* | *DCK* | *CASQ1* | *ENPP4* | *FZD6* |
| *BCAM* | *BACE1* | *DAO* | *GABPB2* | *DCP1A* | *CAVIN2* | *EPHX4* | *G6PC* |
| *BCO1* | *BAG2* | *DAP* | *GALNT9* | *DCTN5* | *CBFA2T2* | *ERGIC2* | *GATM* |
| *BEST4* | *BAG6* | *DCLK1* | *GAREM2* | *DDIT4* | *CBLN1* | *EXOC5* | *GDF11* |
| *BHMT2* | *BAHD1* | *DCP2* | *GDNF* | *DDR1* | *CCDC102A* | *EXOSC1* | *GDF5* |
| *BIN2* | *BAIAP2* | *DCTN2* | *GLI3* | *DDX58* | *CCDC106* | *F2R* | *GFRA1* |
| *BMP1* | *BCKDK* | *DDAH1* | *GLIS3* | *DESI1* | *CCDC25* | *FAM117B* | *GLCCI1* |
| *BRAP* | *BCL7A* | *DDR1* | *GNA13* | *DLAT* | *CCDC32* | *FAM120C* | *GLIS3* |
| *BRD9* | *BEST4* | *DDRGK1* | *GNGT2* | *DLX2* | *CCDC97* | *FAM126A* | *GRIA2* |
| *BRWD3* | *BHLHE41* | *DESI1* | *GPATCH2L* | *DMGDH* | *CCER2* | *FAM167A* | *GRPR* |
| *BTBD19* | *BHMT2* | *DGKG* | *GPR156* | *DNAJB14* | *CCNG2* | *FAM168A* | *GTDC1* |
| *C17orf64* | *BICDL1* | *DGUOK* | *GPT2* | *DPAGT1* | *CCR7* | *FAM169A* | *HAT1* |
| *C19orf12* | *BRD9* | *DHDDS* | *GRAMD2B* | *DPYD* | *CCT7* | *FAM171B* | *HECTD1* |
| *C19orf38* | *BROX* | *DISP3* | *GRIK2* | *DRAXIN* | *CD40LG* | *FAM76B* | *HECTD2* |
| *C1orf115* | *BRPF3* | *DLG3* | *GYG2* | *DYNC1I2* | *CD53* | *FGF10* | *HTR2A* |
| *C1QA* | *BTBD11* | *DNAJC6* | *H6PD* | *DYRK1A* | *CD84* | *FKBP14* | *ICE2* |
| *C1QL1* | *BVES* | *DNER* | *HAPLN4* | *EAF2* | *CDC25B* | *FKBP4* | *IL33* |
| *C1QTNF7* | *C11orf97* | *DPAGT1* | *HCAR1* | *EDEM1* | *CDC42BPA* | *FMO3* | *INSR* |
| *C1QTNF8* | *C18orf25* | *DPH3* | *HDGFL2* | *EFNB3* | *CDC42BPB* | *FNBP1L* | *INTS2* |
| *C20orf194* | *C19orf38* | *DPH6* | *HOXC5* | *EGR1* | *CDC42SE1* | *FOPNL* | *IPO9* |
| *C2CD4C* | *C1QTNF5* | *DPYD* | *HPCAL1* | *EIF1AD* | *CDH8* | *FOXJ3* | *ISL2* |
| *C2orf72* | *C20orf194* | *DRAXIN* | *HSP90AA1* | *ELAVL2* | *CDK12* | *FREM2* | *ITFG1* |
| *C4orf54* | *C20orf27* | *DSTYK* | *ILKAP* | *ELMO2* | *CDK15* | *FRS3* | *JDP2* |
| *C7orf43* | *C2CD4C* | *DUSP1* | *INHBB* | *EEF1A1* | *CDK18* | *FSTL3* | *JMY* |
| *CABLES1* | *C2orf68* | *DYRK2* | *INPP5A* | *EPHA4* | *CDR2L* | *GABRB3* | *KCNJ2* |
| *CACFD1* | *C2orf72* | *EEF1AKMT3* | *INPPL1* | *ERGIC2* | *CELF2* | *GATB* | *KCTD18* |
| *CACNA1G* | *C4orf54* | *EFCAB1* | *ITGAL* | *EXD1* | *CELSR2* | *GDNF* | *KDM1A* |
| *CACNA1H* | *C5orf24* | *EFEMP2* | *ITIH5* | *FAM126A* | *CEMIP* | *GFAP* | *KHSRP* |
| *CACNA1I* | *C5orf51* | *EHBP1* | *JMJD6* | *FAM221A* | *CEP85* | *GKAP1* | *KIAA1024* |
| *CACNB3* | *CA3* | *EHD2* | *KANSL3* | *FAM49B* | *CHAT* | *GLA* | *KLHL15* |
| *CACNG4* | *CAB39* | *EIF5A2* | *KIAA1024* | *FAM53C* | *CHD6* | *GLO1* | *KLHL31* |
| *CALHM3* | *CABLES1* | *ELF4* | *KIAA1324L* | *FBXO7* | *CHERP* | *GLP2R* | *KLK13* |
| *CAPN15* | *CACNA1I* | *EPB41L1* | *KIRREL1* | *FGF10* | *CHFR* | *GNAZ* | *KRT28* |
| *CAPN5* | *CACNB2* | *ERGIC1* | *KMT2A* | *FGF13* | *CHMP1A* | *GPATCH3* | *LIPN* |
| *CARD11* | *CACNB3* | *ERGIC2* | *KMT5B* | *FGL1* | *CHP2* | *GRIA2* | *LNX1* |
| miR-361-3p | **miR-322-5p** | **miR-365-2-5p** | **miR-1247-3p** | **miR-222-5p** | **Novel 137** | **Novel 141** | **Novel 102** |
| *CARNMT1* | *CACNG4* | *EXOC5* | *LAMC1* | *FKBP14* | *CHRDL1* | *GRIK2* | *LPGAT1* |
| *CASK* | *CACUL1* | *EXOG* | *LDLRAD4* | *FLOT2* | *CHRNA1* | *GRIK5* | *LUC7L3* |
| *CASTOR1* | *CALHM5* | *FAM107A* | *LEF1* | *FLT3* | *CHRNA3* | *GRM1* | *MALT1* |
| *CBLN1* | *CAP2* | *FAM173B* | *LMBR1L* | *FOXO3* | *CHST8* | *GSTCD* | *MAML1* |
| *CBX2* | *CAPZA2* | *FAM198B* | *LMNB2* | *FREM2* | *CLCF1* | *HBEGF* | *MAP3K12* |
| *CC2D2A* | *CARM1* | *FAM53C* | *LTBP4* | *FRMD4B* | *CNGA3* | *HCCS* | *MAPK8* |
| *CCDC102A* | *CARNMT1* | *FAM76B* | *MARCH9* | *GALNT7* | *CNNM4* | *HECTD1* | *MBNL1* |
| *CCDC106* | *CASK* | *FBLN2* | *MAX* | *GATA4* | *COBLL1* | *HECTD3* | *MED13* |
| *CCDC137* | *CASKIN2* | *FBXO28* | *MCM4* | *GDA* | *COPS7B* | *HNRNPK* | *MFN1* |
| *CCDC88C* | *CASR* | *FBXO45* | *MFGE8* | *GLI3* | *COPZ1* | *HOXC8* | *MMADHC* |
| *CCHCR1* | *CASZ1* | *FDCSP* | *MFNG* | *GLMN* | *CPLX2* | *HTR2A* | *MMD* |
| *CCNE1* | *CAT* | *FEZ2* | *MIER2* | *GNA12* | *CPTP* | *HTR4* | *MOSPD3* |
| *CCR7* | *CBR3* | *FEZF1* | *MMP13* | *GNAT3* | *CRACR2A* | *HTR5A* | *MTHFD2L* |
| *CCT7* | *CBX2* | *FGB* | *MNT* | *GNPAT* | *CRHR2* | *IDI1* | *NAA15* |
| *CD22* | *CC2D1B* | *FGF10* | *MOB3B* | *GPLD1* | *CRIM1* | *IFNGR1* | *NAXD* |
| *CD68* | *CCDC191* | *FLOT2* | *MPZ* | *GPR158* | *CTDSP1* | *IKZF4* | *NCKAP1L* |
| *CD81* | *CCDC85A* | *FOPNL* | *MSANTD3* | *GRAMD1B* | *CTGF* | *IL17RA* | *NCOA5* |
| *CD9* | *CCDC88C* | *FOXK1* | *MVP* | *GREB1L* | *CTIF* | *IL1RAP* | *NCOR1* |
| *CDC25B* | *CCND2* | *FOXP4* | *NDE1* | *GRIN3A* | *CTNNA1* | *ILF3* | *NDFIP2* |
| *CDC27* | *CCNE1* | *GADD45B* | *NELFE* | *GRK3* | *CTNNBIP1* | *ING5* | *NETO1* |
| *CDC42BPB* | *CCNG2* | *GALNT18* | *NOTCH2* | *GSTCD* | *CTSE* | *INPP4A* | *NNMT* |
| *CDCP1* | *CCNH* | *GALNT3* | *NRBP1* | *GTF2A1* | *CTSF* | *IPO9* | *NSL1* |
| *CDH1* | *CCNJL* | *GATA4* | *NUBP2* | *HAPLN2* | *CTSO* | *KANSL3* | *OSR2* |
| *CDH19* | *CCNL1* | *GDF5* | *NUDT7* | *HERC4* | *CUL4A* | *KAT6B* | *PADI2* |
| *CDIPT* | *CD22* | *GDNF* | *OLFML2A* | *HEY1* | *CXCL13* | *KCNK13* | *PAIP2B* |
| *CDK17* | *CD2AP* | *GLI3* | *PANX1* | *HK1* | *CXCL17* | *KCTD20* | *PANX1* |
| *CDK18* | *CD69* | *GLIS3* | *PAOX* | *HK2* | *CXCL9* | *KDM5A* | *PGM5* |
| *CDKL1* | *CD9* | *GNA13* | *PARD6B* | *HMGCS1* | *CYB5D2* | *KDM7A* | *PICALM* |
| *CDR2L* | *CDC25B* | *GNAO1* | *PAX1* | *HNRNPU* | *CYP26B1* | *KLHL28* | *PID1* |
| *CDS2* | *CDC42SE2* | *GNAT1* | *PAX3* | *HRH4* | *DAAM2* | *KLK13* | *PIK3C2G* |
| *CELF1* | *CDH1* | *GNPAT* | *PCGF3* | *HTR2A* | *DAO* | *KRT28* | *PISD* |
| *CELSR2* | *CDH8* | *GPATCH2L* | *PCNA* | *HTR4* | *DARS* | *KY* | *PJA2* |
| *CEP85* | *CDHR4* | *GPRC5A* | *PEX10* | *ICE2* | *DBH* | *LARP4* | *PKD2* |
| *CERS2* | *CDK12* | *GPT2* | *PGLYRP4* | *IER3* | *DCAKD* | *LDB3* | *PLCXD3* |
| *CERS3* | *CDK17* | *GRK2* | *PGRMC1* | *IFT52* | *DCDC2B* | *LGR4* | *PLEK* |
| *CES1* | *CDK18* | *GSTCD* | *PHGDH* | *IL10* | *DCTN2* | *LHX2* | *PLPP4* |
| *CFAP20* | *CDK5RAP3* | *GSX2* | *PLEKHA8* | *IL17RD* | *DDR1* | *LHX4* | *PNKD* |
| *CGN* | *CDS2* | *HACD4* | *PLXNA2* | *ILF3* | *DEPDC1* | *LMOD3* | *POLK* |
| *CHAD* | *CDX2* | *HAPLN2* | *PPARG* | *ILKAP* | *DGUOK* | *LONRF2* | *PPFIA2* |
| *CHAT* | *CELF1* | *HAPLN4* | *PPP1R15B* | *IMPAD1* | *DHDDS* | *LRCH3* | *PPP1R3A* |
| miR-361-3p | **miR-322-5p** | **miR-365-2-5p** | **miR-1247-3p** | **miR-222-5p** | **Novel 137** | **Novel 141** | **Novel 102** |
| *CHCHD6* | *CEMIP* | *HAUS5* | *PPP2R2C* | *IPO8* | *DHRS7B* | *LRRC18* | *PPP1R8* |
| *CHD5* | *CEP162* | *HBS1L* | *PPP2R2D* | *IRF6* | *DIP2A* | *LRRC73* | *PRDM5* |
| *CHD6* | *CEP85* | *HCCS* | *PPP2R5E* | *ISM1* | *DISP3* | *LZTS3* | *PRKCB* |
| *CHFR* | *CEP85L* | *HES4* | *PRCP* | *ITGA3* | *DLG1* | *MAP3K13* | *PSMC6* |
| *CHKA* | *CERS3* | *HNRNPU* | *PRIMA1* | *ITGAL* | *DLGAP4* | *MARVELD2* | *PSME4* |
| *CHMP1A* | *CHAD* | *HOXC12* | *PRRT1* | *ITPK1* | *DLK1* | *MB21D2* | *PTPRO* |
| *CHMP6* | *CHAF1B* | *HPCAL1* | *PRSS33* | *KAT5* | *DLX3* | *MCHR2* | *QRICH1* |
| *CHMP7* | *CHCHD6* | *HTR4* | *PSD4* | *KCNK9* | *DMBX1* | *MCM7* | *RAB2A* |
| *CHPF* | *CHD5* | *IFNAR2* | *PTGES3L-AARSD1* | *KCTD18* | *DNAJC17* | *MEGF9* | *RAB35* |
| *CHRNA4* | *CHD6* | *IGSF3* | *PTGIR* | *KDM5A* | *DNAJC4* | *MEIS2* | *RBM39* |
| *CHST10* | *CHEK1* | *IL17RA* | *PTK2B* | *KIAA1024* | *DNAJC5* | *METTL13* | *RCOR3* |
| *CHST3* | *CHMP1A* | *ILKAP* | *PTTG1IP* | *KIF5C* | *DNASE1L2* | *MLF2* | *REV3L* |
| *CISH* | *CHMP6* | *INTS8* | *RAP1GAP2* | *KLHL1* | *DOHH* | *MMD* | *RHBDD3* |
| *CKAP4* | *CHMP7* | *IPO13* | *RAX* | *KLHL3* | *DPP6* | *MNT* | *RHOBTB1* |
| *CLCN7* | *CHRNA1* | *IPO9* | *RGL1* | *KNCN* | *DRGX* | *MOSPD1* | *RIOX2* |
| *CLEC17A* | *CHRNA3* | *ITGAL* | *RGMB* | *KPNB1* | *DUS4L* | *MROH2B* | *RNF150* |
| *CLSTN2* | *CHRNA5* | *ITM2C* | *RIMS4* | *LALBA* | *DUSP1* | *MRPL32* | *RRP15* |
| *CLUH* | *CHRNB4* | *JMJD6* | *RPGRIP1L* | *LDB3* | *DUSP8* | *MS4A2* | *RTN4* |
| *CMTR1* | *CHST10* | *JOSD1* | *RPTOR* | *LETM2* | *DVL1* | *MSI1* | *RXFP2* |
| *CNN2* | *CISH* | *KANK4* | *SAMD4B* | *LGI2* | *DVL3* | *MSL1* | *SDHB* |
| *CNOT9* | *CIT* | *KANSL3* | *SBK1* | *LHX4* | *E2F4* | *MSS51* | *SEC24D* |
| *CNTFR* | *CKAP4* | *KARS* | *SCAPER* | *LMLN* | *ECH1* | *MTFP1* | *SEMA4F* |
| *CNTN2* | *CLCN4* | *KAT5* | *SCRN1* | *LMO3* | *EDEM1* | *NABP1* | *SERINC1* |
| *COL26A1* | *CLCN5* | *KCNJ2* | *SDHB* | *LRCH3* | *EEPD1* | *NCALD* | *SF3A1* |
| *COL8A2* | *CLCN7* | *KCTD15* | *SEPHS1* | *LRIG1* | *EFEMP2* | *NCKAP1* | *SFRP1* |
| *COL9A2* | *CLSTN2* | *KDM5A* | *SGCG* | *LRP8* | *EFNA2* | *NENF* | *SGCG* |
| *COMMD2* | *CLUH* | *KHNYN* | *SGK1* | *LRRC18* | *EFR3B* | *NEURL1* | *SGPP1* |
| *COMP* | *CNDP2* | *KIF3B* | *SH3BP4* | *LRRC57* | *EHBP1* | *NFRKB* | *SKAP2* |
| *COPG1* | *CNTN1* | *KIF3C* | *SIAH3* | *LRRC75A* | *EHD2* | *NR1D1* | *SLC16A14* |
| *COPZ1* | *CNTN3* | *KIRREL1* | *SIX5* | *LRRTM2* | *EHD3* | *NRP1* | *SLC25A40* |
| *COQ8A* | *CNTNAP1* | *KLF3* | *SKA3* | *LTBP2* | *ELAVL2* | *NUP35* | *SLC2A12* |
| *CORO6* | *COBLL1* | *KLHDC8A* | *SLC7A6* | *MAK* | *ELF4* | *NXNL2* | *SLC39A8* |
| *CPA5* | *COL8A2* | *KLHL22* | *SNRNP70* | *MANEA* | *ELMO2* | *P3H1* | *SLC5A1* |
| *CPEB3* | *COPS7A* | *KLHL28* | *SPATA33* | *MAP3K12* | *ELOF1* | *PAAF1* | *SLC6A14* |
| *CPLX2* | *COPS7B* | *KLHL42* | *SPSB4* | *MAX* | *EMC1* | *PAIP2B* | *SMCR8* |
| *CPSF7* | *COX6B2* | *KMT5B* | *STAU2* | *MCC* | *EMP2* | *PALB2* | *SNRPB2* |
| *CRTAC1* | *CPA4* | *KNCN* | *STK24* | *MCM7* | *EOGT* | *PDLIM3* | *SNTB2* |
| *CRYBB3* | *CPD* | *KRT32* | *STK40* | *MCTP1* | *EPB41* | *PDLIM4* | *SRP72* |
| *CRYGS* | *CPEB3* | *KRT74* | *STRIP1* | *ME1* | *EPB41L1* | *PEX26* | *SRSF6* |
| *CS* | *CPNE3* | *LEF1* | *SYNGR1* | *MED13* | *EPCAM* | *PGM5* | *ST6GALNAC4* |
| miR-361-3p | **miR-322-5p** | **miR-365-2-5p** | **miR-1247-3p** | **miR-222-5p** | **Novel 137** | **Novel 141** | **Novel 102** |
| *CSF2RB* | *CPSF6* | *LGI1* | *SYT4* | *MED8* | *EPHX1* | *PHF23* | *STAG2* |
| *CSK* | *CPSF7* | *LHX2* | *SYVN1* | *MGARP* | *EPSTI1* | *PHOSPHO1* | *SUPV3L1* |
| *CTBP1* | *CRACR2A* | *LIMCH1* | *TACC1* | *MLF2* | *ERGIC1* | *PIBF1* | *SYNGR3* |
| *CTDSP1* | *CREB3L1* | *LIMK1* | *TBC1D14* | *MMP11* | *ERP44* | *PID1* | *SYT4* |
| *CTDSPL2* | *CRHR2* | *LIN52* | *TBX1* | *MMP2* | *EXO1* | *PIK3AP1* | *TBC1D15* |
| *CTGF* | *CRIM1* | *LMO3* | *THOP1* | *MPP2* | *EXOC1* | *PLA2G4D* | *TBC1D7* |
| *CTIF* | *CSDE1* | *LNX1* | *TIMM17B* | *MPP7* | *F13A1* | *PNPLA5* | *TENM1* |
| *CTNNBL1* | *CTDP1* | *LOC101080954* | *TK1* | *MRPL32* | *FA2H* | *POGK* | *THAP5* |
| *CTSF* | *CTH* | *LOC101086271* | *TMCO4* | *MSANTD3* | *FAIM2* | *POPDC2* | *THRAP3* |
| *CTSV* | *CTIF* | *LOC102901088* | *TMEM38A* | *MSR1* | *FAM107A* | *PRDM2* | *THRB* |
| *CUL4A* | *CTNNBIP1* | *LONRF2* | *TMEM72* | *N4BP2L1* | *FAM120C* | *PRR14L* | *TLK2* |
| *CXCL9* | *CTNNBL1* | *LRFN2* | *TNFAIP3* | *NACC2* | *FAM131C* | *PSAT1* | *TMEM196* |
| *CXCR3* | *CTSF* | *LRP12* | *TNS1* | *NAIF1* | *FAM155B* | *PUM2* | *TMEM26* |
| *CYB5D2* | *CUL4A* | *LRRC49* | *TRAPPC11* | *NAIP* | *FAM167A* | *RAE1* | *TMEM37* |
| *CYP26B1* | *CXCR3* | *LRRC59* | *TRIB2* | *NANS* | *FAM168A* | *RALYL* | *TMEM72* |
| *DAAM2* | *CYB5D2* | *LRRC75A* | *TRIM46* | *NCOA1* | *FAM168B* | *RASSF8* | *TMTC2* |
| *DAZAP1* | *CYB5R1* | *LYNX1* | *TRIP12* | *NDUFAF2* | *FAM213A* | *RBL1* | *TNFAIP3* |
| *DBH* | *CYP26B1* | *MAK* | *TSPAN14* | *NDUFV2* | *FAM216B* | *RBM14* | *TNKS* |
| *DBNDD2* | *CYP39A1* | *MALT1* | *TSPEAR* | *NETO1* | *FAM76A* | *RBM39* | *TNS4* |
| *DCTN2* | *DAZAP1* | *MAP2* | *TTL* | *NETO2* | *FAM83A* | *RFX7* | *TP53INP1* |
| *DDIT4* | *DCLK1* | *MAP3K12* | *TUBB6* | *NIPSNAP2* | *FAM83B* | *RIMKLA* | *TRA2B* |
| *DDR1* | *DCP1A* | *MARVELD2* | *UBASH3A* | *NOX4* | *FAM8A1* | *RNF103* | *TRIM33* |
| *DDRGK1* | *DCP2* | *MAU2* | *UNC13B* | *NR0B1* | *FBLN2* | *RNF170* | *TRIM37* |
| *DDX41* | *DCTN5* | *MCC* | *USH1G* | *NR3C1* | *FBXL18* | *RNF20* | *TRIML1* |
| *DEF8* | *DDRGK1* | *MDFI* | *USP7* | *NRF1* | *FBXO33* | *RPA2* | *TSPEAR* |
| *DERL3* | *DDX3X* | *MDH2* | *VSX2* | *NRIP3* | *FBXO7* | *RTN1* | *TTC13* |
| *DES* | *DDX41* | *MELK* | *WBP2* | *NUCB1* | *FBXW5* | *SAMD5* | *TUB* |
| *DGAT1* | *DDX58* | *MFAP3* | *WDFY1* | *NUDCD3* | *FEZ2* | *SAP30BP* | *UBASH3A* |
| *DGKD* | *DEF8* | *MFGE8* | *WDR5* | *OCIAD1* | *FGF11* | *SAR1A* | *UBE2K* |
| *DIP2A* | *DENND2A* | *MFSD14B* | *WDR59* | *ODF2* | *FLT4* | *SDC4* | *UCHL5* |
| *DLG3* | *DENND2C* | *MFSD9* | *WNT1* | *PAK4* | *FMO4* | *SEMA3D* | *USP53* |
| *DLGAP4* | *DENND4A* | *MIF4GD* | *YY1* | *PALB2* | *FOXF1* | *SGCG* | *WDR20* |
| *DLX3* | *DENND6A* | *MMEL1* | *ZCCHC2* | *PATZ1* | *FOXK1* | *SHCBP1* | *WDR47* |
| *DNAI2* | *DERL3* | *MPZ* | *ZDHHC8* | *PCGF5* | *FOXO4* | *SLC19A2* | *WDR59* |
| *DNAJA4* | *DES* | *MRPL15* | *ZFAT* | *PDE12* | *FOXP4* | *SLC25A18* | *WDR64* |
| *DNAJC16* | *DESI1* | *MRPS10* | *ZIC5* | *PDE3B* | *FRMD8* | *SLC6A14* | *WWC2* |
| *DNAJC17* | *DGAT1* | *MRPS27* | *ZMYND19* | *PDE7B* | *FSTL3* | *SLC8A3* | *WWTR1* |
| *DOK4* | *DGKD* | *MSANTD3* | *ZNF304* | *PDE8A* | *FUT8* | *SNX12* | *ZBTB11* |
| *DOK7* | *DGKQ* | *MSRB3* | *ZNF862* | *PDXK* | *FZD4* | *SNX4* | *ZBTB8A* |
| *DOLPP1* | *DHDDS* | *MSTN* |  | *PDZD7* | *FZD6* | *SPATA2L* | *ZDHHC17* |
| miR-361-3p | **miR-322-5p** | **miR-365-2-5p** | **miR-1247-3p** | **miR-222-5p** | **Novel 137** | **Novel 141** | **Novel 102** |
| *DPAGT1* | *DHTKD1* | *MSX2* |  | *PDZD8* | *G6PC* | *SPTLC2* | *ZFHX3* |
| *DPP6* | *DLG3* | *MVP* |  | *PEG3* | *G6PC3* | *SRI* | *ZIC1* |
| *DPT* | *DLL1* | *MYOC* |  | *PHAX* | *GALNT2* | *SSPN* | *ZNF420* |
| *DPYSL4* | *DLL4* | *MYOZ1* |  | *PID1* | *GALNT9* | *STAG2* | *ZNF768* |
| *DRAP1* | *DNAJA4* | *MYRF* |  | *PIGB* | *GATA2* | *STIP1* | *ZNF790* |
| *DRAXIN* | *DNAJC10* | *NAT9* |  | *PIGK* | *GATAD2A* | *STK10* | *ZSCAN29* |
| *DRGX* | *DNAJC17* | *NDRG2* |  | *PLD1* | *GDF5* | *STRADB* |  |
| *DRP2* | *DNAJC19* | *NDUFA10* |  | *PLEKHG5* | *GDPD2* | *STRIP1* |  |
| *DSTYK* | *DOCK2* | *NDUFB3* |  | *PLPBP* | *GLG1* | *SULF2* |  |
| *DTX1* | *DOK7* | *NEURL1B* |  | *POFUT1* | *GLIS3* | *SV2C* |  |
| *DUOXA1* | *DOLPP1* | *NFKBIE* |  | *POLR2B* | *GLOD5* | *SYNE4* |  |
| *DUS4L* | *DPAGT1* | *NFRKB* |  | *POLR3F* | *GLS2* | *TATDN1* |  |
| *DUSP8* | *DPP6* | *NGLY1* |  | *PPARG* | *GNA12* | *TBC1D15* |  |
| *DVL1* | *DRAM1* | *NIFK* |  | *PPP1R17* | *GNAZ* | *TBC1D23* |  |
| *DYM* | *DRAXIN* | *NIPSNAP2* |  | *PPP2R5E* | *GNGT2* | *TBCCD1* |  |
| *E2F4* | *DRD1* | *NKD1* |  | *PRDM10* | *GNL2* | *TENT4A* |  |
| *EBF4* | *DSTYK* | *NMRK1* |  | *PRPF19* | *GOLGA1* | *TERB2* |  |
| *ECH1* | *DTX4* | *NOTCH2* |  | *PRPSAP2* | *GPC4* | *THAP6* |  |
| *ECI1* | *DUSP1* | *NR1D1* |  | *PTCH1* | *GPR156* | *THEMIS* |  |
| *EDEM1* | *DUSP8* | *NRF1* |  | *PTCHD1* | *GPR157* | *THNSL2* |  |
| *EEPD1* | *DYNC1LI2* | *NRN1L* |  | *PTGER2* | *GPR179* | *TIGAR* |  |
| *EFCC1* | *DYRK2* | *NSG2* |  | *PUM2* | *GPR83* | *TIMP4* |  |
| *EFEMP2* | *DZIP1* | *NUDCD1* |  | *PWWP2A* | *GRAMD1B* | *TMPRSS11F* |  |
| *EFNA2* | *ECEL1* | *NUDCD3* |  | *RAD51D* | *GRB7* | *TNFAIP3* |  |
| *EFNB3* | *EFCC1* | *ODF1* |  | *RALGAPA2* | *GRIK1* | *TOP2B* |  |
| *EFR3B* | *EFEMP2* | *ODF2* |  | *RAPGEF4* | *GRIN3A* | *TOPORS* |  |
| *EHBP1* | *EIF2B5* | *OLR1* |  | *RARS* | *GRPR* | *TOR2A* |  |
| *EHD2* | *EIF4G1* | *OSBPL2* |  | *RASD1* | *GTF3C1* | *TRIM2* |  |
| *EHD4* | *EIF5A2* | *PAFAH1B2* |  | *RASD2* | *HAPLN2* | *TTC13* |  |
| *EHMT1* | *ELF4* | *PCDH17* |  | *RB1* | *HAT1* | *U2SURP* |  |
| *EIF1AD* | *ELF5* | *PCDHGC5* |  | *RCN2* | *HAUS3* | *UBA5* |  |
| *EIF4A3* | *ELMO2* | *PCGF2* |  | *RGMB* | *HK1* | *UBE2J1* |  |
| *EIF4EBP1* | *ELOVL5* | *PCSK7* |  | *RGS7BP* | *HNRNPU* | *UBTD2* |  |
| *EIF4G1* | *ELOVL6* | *PCYOX1* |  | *RHOBTB1* | *HOXC5* | *UCP3* |  |
| *ELF4* | *ELP1* | *PDCD1LG2* |  | *RMDN3* | *HS1BP3* | *UHRF1BP1* |  |
| *ELMOD2* | *EML4* | *PDE2A* |  | *ROGDI* | *HSD11B2* | *UPB1* |  |
| *ELP3* | *ENTPD7* | *PDE3B* |  | *RPL15* | *HTR2C* | *USH1G* |  |
| *ENSA* | *ENTR1* | *PDE6H* |  | *RRP15* | *HTR6* | *WDFY1* |  |
| *ENTPD2* | *EPB41L1* | *PDGFRB* |  | *RSPH4A* | *IFITM5* | *WDR35* |  |
| *ENTPD6* | *EPB41L5* | *PDS5A* |  | *SALL3* | *IGF2BP1* | *WRNIP1* |  |
| miR-361-3p | **miR-322-5p** | **miR-365-2-5p** | **miR-1247-3p** | **miR-222-5p** | **Novel 137** | **Novel 141** | **Novel 102** |
| *EPB41* | *EPC1* | *PDXK* |  | *SDC2* | *IGFBP5* | *XPNPEP1* |  |
| *EPHA4* | *EPSTI1* | *PDZD7* |  | *SERPINB12* | *IGSF8* | *YY1* |  |
| *ERLIN2* | *ETV3* | *PDZK1* |  | *SERPINB7* | *IL13RA1* | *ZC3H12B* |  |
| *ERRFI1* | *EXO1* | *PHC2* |  | *SERPINE2* | *IL1RN* | *ZDHHC15* |  |
| *ETV3* | *EXOC1* | *PHLDB1* |  | *SERTAD4* | *ILF3* | *ZIC2* |  |
| *ETV4* | *EXOC6* | *PI3* |  | *SF3A1* | *IMPDH1* | *ZIC3* |  |
| *EXOC1* | *EXTL2* | *PI4KB* |  | *SH3RF2* | *ING4* | *ZKSCAN7* |  |
| *FAAP24* | *EYA1* | *PIANP* |  | *SKA3* | *INTS10* | *ZNF182* |  |
| *FABP6* | *FAIM* | *PIBF1* |  | *SLC13A1* | *IP6K3* | *ZNF207* |  |
| *FAIM2* | *FAM120C* | *PLD5* |  | *SLC25A30* | *IRX6* | *ZNF365* |  |
| *FAM102A* | *FAM126A* | *PLEKHS1* |  | *SLC2A12* | *ISCA1* | *ZNF512* |  |
| *FAM107A* | *FAM155B* | *PLPBP* |  | *SLC6A14* | *ISL1* | *ZPBP* |  |
| *FAM120C* | *FAM160B2* | *PLPP4* |  | *SLC9A4* | *ITGA10* |  |  |
| *FAM129B* | *FAM163A* | *PNPLA5* |  | *SLCO4C1* | *ITGAL* |  |  |
| *FAM131C* | *FAM193A* | *POU2F2* |  | *SLX4IP* | *ITPK1* |  |  |
| *FAM168A* | *FAM196B* | *PPAT* |  | *SMOC1* | *JADE2* |  |  |
| *FAM193A* | *FAM69A* | *PPP1R17* |  | *SORT1* | *JMJD6* |  |  |
| *FAM193B* | *FAM83F* | *PPP1R8* |  | *SPATA19* | *JPH4* |  |  |
| *FAM198B* | *FAM89B* | *PPP1R9B* |  | *SPATA33* | *JUP* |  |  |
| *FAM219B* | *FBLN2* | *PPP2R1A* |  | *ST7L* | *KANSL1* |  |  |
| *FAM3C* | *FBXL18* | *PPP2R2B* |  | *STIP1* | *KANSL3* |  |  |
| *FAM57B* | *FBXL20* | *PRDM10* |  | *STK17B* | *KAT6A* |  |  |
| *FAM71F2* | *FBXO17* | *PRDM2* |  | *STMN2* | *KAT6B* |  |  |
| *FAM83A* | *FBXO28* | *PRKRA* |  | *STRIP1* | *KAT7* |  |  |
| *FAM83F* | *FBXO45* | *PSCA* |  | *SUOX* | *KCNC4* |  |  |
| *FAM89B* | *FBXW4* | *PSD4* |  | *SV2C* | *KCNK9* |  |  |
| *FBXL18* | *FDXR* | *PSKH1* |  | *SYNJ2BP* | *KCNN4* |  |  |
| *FBXO28* | *FERMT1* | *PTGIR* |  | *SYT9* | *KCTD15* |  |  |
| *FBXO44* | *FGF20* | *PUF60* |  | *TAB2* | *KDM5C* |  |  |
| *FBXW2* | *FGFR1* | *PUM2* |  | *TAF2* | *KDM7A* |  |  |
| *FBXW5* | *FGFR4* | *PYROXD2* |  | *TAGAP* | *KDR* |  |  |
| *FBXW8* | *FGGY* | *R3HDM2* |  | *TBC1D14* | *KIF2C* |  |  |
| *FERMT1* | *FKBP4* | *RABGAP1L* |  | *TGFBR3* | *KIF3B* |  |  |
| *FGF23* | *FKBP5* | *RAPGEF4* |  | *TIMP4* | *KIF5B* |  |  |
| *FGFR1* | *FLOT2* | *RASAL1* |  | *TIPRL* | *KIFC3* |  |  |
| *FGFR4* | *FLT3* | *RASEF* |  | *TLE4* | *KIRREL1* |  |  |
| *FKBP4* | *FMNL3* | *RASGEF1B* |  | *TM2D3* | *KLHDC8A* |  |  |
| *FLNB* | *FOPNL* | *RBFOX1* |  | *TMED10* | *KLHL22* |  |  |
| *FLT4* | *FOXA2* | *RBM18* |  | *TMEM106B* | *KLHL23* |  |  |
| *FOPNL* | *FOXK1* | *RBM6* |  | *TMEM123* | *KLHL26* |  |  |
| *FOXA1* | *FPGT* | *RCC1L* |  | *TMEM168* | *KRT4* |  |  |
| miR-361-3p | **miR-322-5p** | **miR-365-2-5p** | **miR-1247-3p** | **miR-222-5p** | **Novel 137** | **Novel 141** | **Novel 102** |
| *FOXF1* | *FRMPD1* | *RDH10* |  | *TMEM196* | *KRT72* |  |  |
| *FOXJ1* | *FRYL* | *RET* |  | *TMEM248* | *KRT74* |  |  |
| *FOXJ3* | *FSD1* | *RGMA* |  | *TMEM47* | *KRTCAP2* |  |  |
| *FOXP4* | *FSTL3* | *RGN* |  | *TMEM52B* | *LAMC1* |  |  |
| *FOXRED1* | *FZD4* | *RIMS4* |  | *TMEM62* | *LARP4* |  |  |
| *FRMD8* | *FZD6* | *RNF170* |  | *TMOD1* | *LATS1* |  |  |
| *FXYD1* | *GABRE* | *RNF43* |  | *TMPRSS11E* | *LBP* |  |  |
| *FZD4* | *GABRG2* | *ROGDI* |  | *TNFRSF11B* | *LDHC* |  |  |
| *G6PC* | *GALNT14* | *RPP30* |  | *TNMD* | *LEF1* |  |  |
| *GABPB2* | *GALNT3* | *RPRD1B* |  | *TOPBP1* | *LGI1* |  |  |
| *GADD45B* | *GALNT7* | *RPS13* |  | *TOR1B* | *LGI2* |  |  |
| *GALE* | *GALNT9* | *RPS6KA3* |  | *TP53INP1* | *LGR6* |  |  |
| *GALM* | *GAS2L3* | *RUBCN* |  | *TPM3* | *LHX4* |  |  |
| *GALNT18* | *GATA4* | *RUFY1* |  | *TRA2B* | *LIN9* |  |  |
| *GALNT9* | *GATAD2A* | *RXRA* |  | *TRIM2* | *LMNB2* |  |  |
| *GATA2* | *GCH1* | *SAMD14* |  | *TRIM33* | *LMX1B* |  |  |
| *GATA4* | *GDA* | *SAMD4B* |  | *TRIM37* | *LONRF2* |  |  |
| *GATAD2A* | *GDF5* | *SBSPON* |  | *TSPAN14* | *LRCH2* |  |  |
| *GCDH* | *GDPD2* | *SCAMP4* |  | *TUB* | *LRCH4* |  |  |
| *GDE1* | *GFAP* | *SCGB1A1* |  | *TUBGCP4* | *LRRC52* |  |  |
| *GDF11* | *GFI1B* | *SDC4* |  | *TWISTNB* | *LTBP2* |  |  |
| *GDPD1* | *GID8* | *SEC61A1* |  | *U2SURP* | *LYPLA2* |  |  |
| *GJB4* | *GIGYF1* | *SERINC1* |  | *UBE2H* | *LZTS3* |  |  |
| *GLG1* | *GJB4* | *SERPINB5* |  | *UBE3D* | *MAN1C1* |  |  |
| *GLI2* | *GLDN* | *SFPQ* |  | *UBR2* | *MAP2* |  |  |
| *GLIS2* | *GLG1* | *SIDT1* |  | *UNC119B* | *MAP2K3* |  |  |
| *GLIS3* | *GLIS3* | *SKAP2* |  | *USP14* | *MAP3K13* |  |  |
| *GLOD5* | *GLOD5* | *SLC16A9* |  | *WDHD1* | *MAP9* |  |  |
| *GNA12* | *GLS2* | *SLC17A8* |  | *WDR47* | *MAPK10* |  |  |
| *GNA13* | *GLYCTK* | *SLC25A18* |  | *WDR59* | *MATN4* |  |  |
| *GNAT1* | *GNA13* | *SLC25A30* |  | *WNT4* | *MBD6* |  |  |
| *GNL2* | *GNAL* | *SLC35E4* |  | *WNT9A* | *MCM7* |  |  |
| *GNMT* | *GNAO1* | *SLC39A3* |  | *XPO7* | *MCRS1* |  |  |
| *GNPAT* | *GNAT1* | *SLC5A1* |  | *ZCCHC10* | *MDH2* |  |  |
| *GOLGA7B* | *GNAZ* | *SLC6A2* |  | *ZDHHC15* | *MED13* |  |  |
| *GPATCH2L* | *GNMT* | *SLC6A6* |  | *ZER1* | *MED8* |  |  |
| *GPC1* | *GOLGA1* | *SLC9A4* |  | *ZFHX2* | *MEIS1* |  |  |
| *GPC2* | *GOLGA7B* | *SLCO2B1* |  | *ZIC3* | *METTL3* |  |  |
| *GPC4* | *GPR162* | *SMAD3* |  | *ZKSCAN8* | *METTL7B* |  |  |
| *GPD1* | *GPRC5D* | *SMOC2* |  | *ZMAT4* | *MFAP5* |  |  |
| *GPLD1* | *GPT2* | *SNAP29* |  | *ZNF395* | *MKL2* |  |  |
| miR-361-3p | **miR-322-5p** | **miR-365-2-5p** | **miR-1247-3p** | **miR-222-5p** | **Novel 137** | **Novel 141** | **Novel 102** |
| *GPR156* | *GRID2* | *SNRPA* |  | *ZNF596* | *MLC1* |  |  |
| *GPRC5A* | *GRIN3A* | *SNTB1* |  | *ZNF598* | *MLF2* |  |  |
| *GPRC6A* | *GRM1* | *SPATA45* |  | *ZNF629* | *MMGT1* |  |  |
| *GPS1* | *GSE1* | *SPIB* |  | *ZNF768* | *MMP14* |  |  |
| *GPT* | *GSKIP* | *SPIDR* |  | *ZNF879* | *MMP25* |  |  |
| *GPT2* | *GSTA4* | *SRI* |  |  | *MNT* |  |  |
| *GRIK5* | *GSTT4* | *STX11* |  |  | *MOB3B* |  |  |
| *GRIN3A* | *GTDC1* | *SULT4A1* |  |  | *MOB3C* |  |  |
| *GRK2* | *GTF3C5* | *SURF4* |  |  | *MPP2* |  |  |
| *GRK3* | *H6PD* | *SUSD6* |  |  | *MPZ* |  |  |
| *GSE1* | *HAPLN2* | *SV2C* |  |  | *MRPL11* |  |  |
| *GTF3C1* | *HAS2* | *SYCN* |  |  | *MRPL32* |  |  |
| *H6PD* | *HAUS2* | *SYNCRIP* |  |  | *MRPL39* |  |  |
| *HAPLN2* | *HDAC6* | *SYNDIG1L* |  |  | *MRPL9* |  |  |
| *HAT1* | *HDLBP* | *TACO1* |  |  | *MRPS10* |  |  |
| *HCAR1* | *HECTD1* | *TBC1D19* |  |  | *MRRF* |  |  |
| *HCRTR1* | *HELZ* | *TBC1D2* |  |  | *MSI1* |  |  |
| *HDAC11* | *HEPHL1* | *TBC1D5* |  |  | *MSN* |  |  |
| *HDAC6* | *HES4* | *TBL2* |  |  | *MSR1* |  |  |
| *HDAC8* | *HK1* | *TCP11L2* |  |  | *MSX1* |  |  |
| *HDGFL2* | *HK2* | *THEMIS* |  |  | *MTFP1* |  |  |
| *HDLBP* | *HKDC1* | *THRAP3* |  |  | *MVP* |  |  |
| *HEPACAM* | *HNRNPU* | *THRB* |  |  | *MX1* |  |  |
| *HES4* | *HOXA10* | *TIMM8A* |  |  | *MXD4* |  |  |
| *HIPK1* | *HOXC8* | *TIMM8A* |  |  | *MYH9* |  |  |
| *HK1* | *HOXD1* | *TMEM106A* |  |  | *MYL6B* |  |  |
| *HNF1B* | *HS3ST3B1* | *TMEM107* |  |  | *MYL9* |  |  |
| *HNRNPR* | *HSPA12B* | *TMEM108* |  |  | *MYO1C* |  |  |
| *HNRNPU* | *HTR2A* | *TMEM150C* |  |  | *MYO3B* |  |  |
| *HOMEZ* | *HTR2C* | *TMEM151A* |  |  | *MYOM3* |  |  |
| *HOXA10* | *HTR4* | *TMEM241* |  |  | *MYOZ1* |  |  |
| *HOXC4* | *HTR6* | *TMEM64* |  |  | *NAGK* |  |  |
| *HOXC5* | *IFIH1* | *TMEM72* |  |  | *NAGLU* |  |  |
| *HOXC8* | *IFT27* | *TNFAIP3* |  |  | *NAT9* |  |  |
| *HS1BP3* | *IFT57* | *TNFAIP6* |  |  | *NATD1* |  |  |
| *HSPA12B* | *IGF2R* | *TNMD* |  |  | *NCAPH* |  |  |
| *HTR2A* | *IGFBP5* | *TPI1* |  |  | *NCOR1* |  |  |
| *HTR6* | *IKBKB* | *TPP2* |  |  | *NDNF* |  |  |
| *HTRA1* | *IKZF2* | *TPRA1* |  |  | *NDRG2* |  |  |
| *IARS* | *IL17RA* | *TRIB3* |  |  | *NDRG4* |  |  |
| *ICK* | *IL1R2* | *TRIM33* |  |  | *NDUFA10* |  |  |
| miR-361-3p | **miR-322-5p** | **miR-365-2-5p** | **miR-1247-3p** | **miR-222-5p** | **Novel 137** | **Novel 141** | **Novel 102** |
| *IFT57* | *IL1RAP* | *TRIP4* |  |  | *NECTIN2* |  |  |
| *IGF2* | *ILF2* | *TRIQK* |  |  | *NENF* |  |  |
| *IGF2R* | *ILF3* | *TRPV5* |  |  | *NEURL1* |  |  |
| *IGFBP5* | *ILKAP* | *TTC13* |  |  | *NEURL1B* |  |  |
| *IGSF3* | *IMPDH1* | *U2SURP* |  |  | *NFRKB* |  |  |
| *IK* | *ING5* | *UBE2J1* |  |  | *NKD1* |  |  |
| *IKZF4* | *INHBB* | *UBL4A* |  |  | *NKTR* |  |  |
| *IL11* | *INSR* | *UBR3* |  |  | *NLN* |  |  |
| *IL13* | *IPO7* | *UBR7* |  |  | *NMNAT2* |  |  |
| *IL31RA* | *IPO8* | *UFM1* |  |  | *NMUR1* |  |  |
| *IL4R* | *IPO9* | *UGCG* |  |  | *NNMT* |  |  |
| *ILKAP* | *ISL1* | *UNC13B* |  |  | *NODAL* |  |  |
| *IMPDH1* | *ISL2* | *URB2* |  |  | *NPPC* |  |  |
| *INAVA* | *ISOC1* | *UROC1* |  |  | *NPTX2* |  |  |
| *ING4* | *ITGA10* | *USP11* |  |  | *NR1I2* |  |  |
| *INPPL1* | *ITGA3* | *VPS72* |  |  | *NR2F1* |  |  |
| *IPO8* | *ITGAL* | *VWA7* |  |  | *NR2F2* |  |  |
| *IRF6* | *JADE2* | *WASF2* |  |  | *NR4A1* |  |  |
| *IRX1* | *JAK2* | *WBP1L* |  |  | *NR5A2* |  |  |
| *IRX6* | *JOSD1* | *WDR26* |  |  | *NRGN* |  |  |
| *ITM2C* | *JPH1* | *WDR7* |  |  | *NSG1* |  |  |
| *ITPK1* | *JPT1* | *WFDC9* |  |  | *NUAK1* |  |  |
| *JAG1* | *KAT8* | *WNT3* |  |  | *NUP153* |  |  |
| *JAZF1* | *KATNB1* | *WNT4* |  |  | *NUP210* |  |  |
| *JMJD6* | *KCNC4* | *WWC1* |  |  | *NXPH2* |  |  |
| *JPH4* | *KCNG1* | *XPO6* |  |  | *OAF* |  |  |
| *JUP* | *KCNH3* | *YME1L1* |  |  | *OLFM1* |  |  |
| *KANK2* | *KCNH4* | *YPEL2* |  |  | *OLFML2A* |  |  |
| *KANK4* | *KCNJ14* | *YTHDF1* |  |  | *OPRD1* |  |  |
| *KANSL1* | *KCNJ2* | *ZBTB21* |  |  | *OPRL1* |  |  |
| *KANSL1L* | *KCNK13* | *ZBTB47* |  |  | *OTUD3* |  |  |
| *KARS* | *KCNN4* | *ZC3H12B* |  |  | *OXSR1* |  |  |
| *KAT5* | *KDM1A* | *ZC3H4* |  |  | *P2RX3* |  |  |
| *KAT7* | *KDM5A* | *ZCCHC24* |  |  | *PAK4* |  |  |
| *KCMF1* | *KDM7A* | *ZDHHC15* |  |  | *PALM* |  |  |
| *KCNC4* | *KDR* | *ZFC3H1* |  |  | *PAX2* |  |  |
| *KCNH2* | *KHNYN* | *ZFP36L1* |  |  | *PCDH17* |  |  |
| *KCNJ2* | *KIAA1522* | *ZIC2* |  |  | *PCDHGC5* |  |  |
| *KCNK12* | *KIF1B* | *ZIC3* |  |  | *PCP4L1* |  |  |
| *KCNK13* | *KIF21A* | *ZMAT4* |  |  | *PCSK5* |  |  |
| miR-361-3p | **miR-322-5p** | **miR-365-2-5p** | **miR-1247-3p** | **miR-222-5p** | **Novel 137** | **Novel 141** | **Novel 102** |
| *KCNK9* | *KIF3B* | *ZNF174* |  |  | *PDE1C* |  |  |
| *KCNN4* | *KIF5B* | *ZNF397* |  |  | *PDGFB* |  |  |
| *KCNQ1* | *KIF5C* | *ZNF48* |  |  | *PDK2* |  |  |
| *KCTD15* | *KIFC3* | *ZNF652* |  |  | *PDLIM4* |  |  |
| *KDM5C* | *KLC2* |  |  |  | *PEX10* |  |  |
| *KDM7A* | *KLHDC8B* |  |  |  | *PEX14* |  |  |
| *KHSRP* | *KLHL15* |  |  |  | *PHF1* |  |  |
| *KIAA0513* | *KLHL18* |  |  |  | *PHF6* |  |  |
| *KIF16B* | *KLHL22* |  |  |  | *PHLDB1* |  |  |
| *KIF1B* | *KLHL23* |  |  |  | *PI4KB* |  |  |
| *KIF3B* | *KLHL26* |  |  |  | *PIANP* |  |  |
| *KIF5C* | *KLHL28* |  |  |  | *PIGB* |  |  |
| *KIRREL1* | *KLHL31* |  |  |  | *PIK3C2G* |  |  |
| *KLC3* | *KMT2A* |  |  |  | *PIP4K2B* |  |  |
| *KLF16* | *KPNB1* |  |  |  | *PISD* |  |  |
| *KLHL17* | *KRT1* |  |  |  | *PITPNM2* |  |  |
| *KLHL22* | *KRT39* |  |  |  | *PKNOX1* |  |  |
| *KLHL3* | *KRT74* |  |  |  | *PLAGL2* |  |  |
| *KRT14* | *KY* |  |  |  | *PLCXD3* |  |  |
| *KRTCAP2* | *LAMC1* |  |  |  | *PLEKHA8* |  |  |
| *KXD1* | *LATS1* |  |  |  | *PLEKHB1* |  |  |
| *KY* | *LCLAT1* |  |  |  | *PLEKHD1* |  |  |
| *LAMP2* | *LDB3* |  |  |  | *PLEKHM2* |  |  |
| *LAT* | *LDLRAD2* |  |  |  | *PLK2* |  |  |
| *LBP* | *LDLRAD4* |  |  |  | *PLXNA1* |  |  |
| *LBX1* | *LEMD2* |  |  |  | *PNCK* |  |  |
| *LDLR* | *LGI2* |  |  |  | *PNKD* |  |  |
| *LDLRAD2* | *LGI3* |  |  |  | *PNPLA5* |  |  |
| *LDLRAD4* | *LGR5* |  |  |  | *PODN* |  |  |
| *LGI2* | *LHX4* |  |  |  | *POFUT1* |  |  |
| *LGI3* | *LIMK1* |  |  |  | *POLK* |  |  |
| *LIMK2* | *LIN52* |  |  |  | *POLR1E* |  |  |
| *LMBR1L* | *LMBR1L* |  |  |  | *POLR2C* |  |  |
| *LMBRD1* | *LMNA* |  |  |  | *POLR3D* |  |  |
| *LMLN* | *LMO7* |  |  |  | *PPARD* |  |  |
| *LMX1B* | *LNX1* |  |  |  | *PPARG* |  |  |
| *LONRF1* | *LOC101083017* |  |  |  | *PPARGC1A* |  |  |
| *LOXL2* | *LONRF2* |  |  |  | *PPIL2* |  |  |
| *LPGAT1* | *LRCH2* |  |  |  | *PPIL3* |  |  |
| *LRP1* | *LRCH3* |  |  |  | *PPP1R15B* |  |  |
| miR-361-3p | **miR-322-5p** | **miR-365-2-5p** | **miR-1247-3p** | **miR-222-5p** | **Novel 137** | **Novel 141** | **Novel 102** |
| *LRRC49* | *LRIG1* |  |  |  | *PPP1R16B* |  |  |
| *LRRC52* | *LRP1* |  |  |  | *PPP1R1B* |  |  |
| *LRRC75A* | *LRRC41* |  |  |  | *PPP1R9B* |  |  |
| *LRTM1* | *LRRC49* |  |  |  | *PPP2R1A* |  |  |
| *LRTM2* | *LRRTM2* |  |  |  | *PPP2R2C* |  |  |
| *LRWD1* | *LRTM2* |  |  |  | *PRDM2* |  |  |
| *LSAMP* | *LRWD1* |  |  |  | *PREP* |  |  |
| *LUZP1* | *LSAMP* |  |  |  | *PRIMA1* |  |  |
| *LY6D* | *LSS* |  |  |  | *PRKAB2* |  |  |
| *LY6E* | *LTBP2* |  |  |  | *PRKAG1* |  |  |
| *LY6H* | *LUZP1* |  |  |  | *PRKAR2A* |  |  |
| *LYPLA2* | *LYPLA2* |  |  |  | *PRKCG* |  |  |
| *LZTS3* | *LZTS3* |  |  |  | *PRKCQ* |  |  |
| *MALT1* | *MAMSTR* |  |  |  | *PSCA* |  |  |
| *MAN2A1* | *MAN2A1* |  |  |  | *PSME4* |  |  |
| *MANF* | *MAP2K1* |  |  |  | *PSORS1C2* |  |  |
| *MAOB* | *MAP2K3* |  |  |  | *PTCD2* |  |  |
| *MAP2* | *MAP3K21* |  |  |  | *PTGES* |  |  |
| *MAP2K3* | *MAPK10* |  |  |  | *PWWP2A* |  |  |
| *MAP2K5* | *MAPK11* |  |  |  | *R3HDM2* |  |  |
| *MAP2K7* | *MAPK1IP1L* |  |  |  | *RABIF* |  |  |
| *MAP3K12* | *MAPK8* |  |  |  | *RALGAPA2* |  |  |
| *MAP3K13* | *MARCH5* |  |  |  | *RALGPS2* |  |  |
| *MAP3K9* | *MARHC10* |  |  |  | *RAP1GAP2* |  |  |
| *MAPK12* | *MAU2* |  |  |  | *RASAL1* |  |  |
| *MAPK3* | *MBNL2* |  |  |  | *RBM18* |  |  |
| *MAPK9* | *MCM7* |  |  |  | *RBM39* |  |  |
| *MAPKAPK3* | *MCOLN1* |  |  |  | *RBPJL* |  |  |
| *MARCH1* | *MCRIP1* |  |  |  | *RGMA* |  |  |
| *MARK4* | *MDH2* |  |  |  | *RGS7BP* |  |  |
| *MATN4* | *ME1* |  |  |  | *RHOBTB2* |  |  |
| *MAU2* | *MED13* |  |  |  | *RIC8A* |  |  |
| *MB* | *MED14* |  |  |  | *RIC8B* |  |  |
| *MBD6* | *MEF2A* |  |  |  | *RNF185* |  |  |
| *MCAT* | *MEGF10* |  |  |  | *RNF2* |  |  |
| *MCM2* | *MEOX2* |  |  |  | *RNPEP* |  |  |
| *MCM3* | *METTL26* |  |  |  | *RPAIN* |  |  |
| *MCM4* | *MFAP5* |  |  |  | *RPL15* |  |  |
| *MCM7* | *MFN2* |  |  |  | *RPL8* |  |  |
| miR-361-3p | **miR-322-5p** | **miR-365-2-5p** | **miR-1247-3p** | **miR-222-5p** | **Novel 137** | **Novel 141** | **Novel 102** |
| *MCRIP1* | *MGRN1* |  |  |  | *RPP30* |  |  |
| *MDH2* | *MICU1* |  |  |  | *RS1* |  |  |
| *MED18* | *MINDY4* |  |  |  | *RSPO2* |  |  |
| *MEF2D* | *MIPOL1* |  |  |  | *RTN4RL1* |  |  |
| *METTL14* | *MKRN2* |  |  |  | *SALL1* |  |  |
| *METTL26* | *MLF2* |  |  |  | *SAMD10* |  |  |
| *MFGE8* | *MMD* |  |  |  | *SBK1* |  |  |
| *MFN2* | *MMGT1* |  |  |  | *SCAMP4* |  |  |
| *MGRN1* | *MMP2* |  |  |  | *SCNM1* |  |  |
| *MIER2* | *MMP25* |  |  |  | *SCNN1G* |  |  |
| *MINDY4* | *MMRN2* |  |  |  | *SCRN1* |  |  |
| *MIP* | *MNT* |  |  |  | *SDHB* |  |  |
| *MISP* | *MOB3B* |  |  |  | *SDR9C7* |  |  |
| *MKL2* | *MORN4* |  |  |  | *SEC61A1* |  |  |
| *MLC1* | *MPPED1* |  |  |  | *SEH1L* |  |  |
| *MLF2* | *MRPL54* |  |  |  | *SEPTIN9* |  |  |
| *MMAB* | *MSL1* |  |  |  | *SERPINB7* |  |  |
| *MMEL1* | *MTFP1* |  |  |  | *SERPINB9* |  |  |
| *MMP14* | *MTFR1L* |  |  |  | *SFRP1* |  |  |
| *MMP16* | *MTMR11* |  |  |  | *SFRP5* |  |  |
| *MNT* | *MYPN* |  |  |  | *SGCB* |  |  |
| *MOV10* | *MYT1L* |  |  |  | *SH3BP2* |  |  |
| *MPND* | *NACC2* |  |  |  | *SIX3* |  |  |
| *MPP2* | *NAGK* |  |  |  | *SKAP2* |  |  |
| *MPPED1* | *NAIF1* |  |  |  | *SLC12A3* |  |  |
| *MPZ* | *NAPB* |  |  |  | *SLC13A1* |  |  |
| *MRC2* | *NAPG* |  |  |  | *SLC17A9* |  |  |
| *MROH2B* | *NAT9* |  |  |  | *SLC25A25* |  |  |
| *MRPL11* | *NATD1* |  |  |  | *SLC25A28* |  |  |
| *MRPL54* | *NCAPG* |  |  |  | *SLC25A30* |  |  |
| *MSR1* | *NCAPH* |  |  |  | *SLC25A35* |  |  |
| *MSX1* | *NCKAP5L* |  |  |  | *SLC26A4* |  |  |
| *MSX2* | *NCOA5* |  |  |  | *SLC29A2* |  |  |
| *MTFP1* | *NDRG4* |  |  |  | *SLC38A3* |  |  |
| *MUL1* | *NDUFA10* |  |  |  | *SLC41A1* |  |  |
| *MVP* | *NECAP2* |  |  |  | *SLC45A3* |  |  |
| *MX1* | *NECTIN2* |  |  |  | *SLC5A7* |  |  |
| *MXD4* | *NEURL1B* |  |  |  | *SLC6A6* |  |  |
| *MYDGF* | *NFATC2* |  |  |  | *SLC7A7* |  |  |
| *MYLK2* | *NFRKB* |  |  |  | *SLC9A3R2* |  |  |
| miR-361-3p | **miR-322-5p** | **miR-365-2-5p** | **miR-1247-3p** | **miR-222-5p** | **Novel 137** | **Novel 141** | **Novel 102** |
| *MYO1C* | *NFYA* |  |  |  | *SLC9A9* |  |  |
| *MYO1D* | *NHS* |  |  |  | *SLCO2B1* |  |  |
| *MYPN* | *NIPAL3* |  |  |  | *SMOC1* |  |  |
| *NAB2* | *NKD1* |  |  |  | *SMPD3* |  |  |
| *NACC2* | *NKTR* |  |  |  | *SMTN* |  |  |
| *NAGLU* | *NLE1* |  |  |  | *SNAI1* |  |  |
| *NAIF1* | *NLN* |  |  |  | *SNAP29* |  |  |
| *NANS* | *NMNAT2* |  |  |  | *SNAPC3* |  |  |
| *NAT8L* | *NMUR2* |  |  |  | *SNRK* |  |  |
| *NAT9* | *NOC4L* |  |  |  | *SORD* |  |  |
| *NATD1* | *NOL3* |  |  |  | *SORL1* |  |  |
| *NCOA3* | *NOTCH2* |  |  |  | *SORT1* |  |  |
| *NDE1* | *NPFFR2* |  |  |  | *SPA17* |  |  |
| *NDUFA10* | *NPPC* |  |  |  | *SPATA2L* |  |  |
| *NELFB* | *NR2F1* |  |  |  | *SPRYD3* |  |  |
| *NELFCD* | *NR4A1* |  |  |  | *SREBF2* |  |  |
| *NEURL1* | *NRBP1* |  |  |  | *SRF* |  |  |
| *NEURL1B* | *NRN1L* |  |  |  | *SRSF10* |  |  |
| *NFKBIE* | *NSG1* |  |  |  | *SSR1* |  |  |
| *NGEF* | *NSUN2* |  |  |  | *STAT4* |  |  |
| *NGLY1* | *NUAK1* |  |  |  | *STK40* |  |  |
| *NHS* | *NUBPL* |  |  |  | *STX11* |  |  |
| *NIPAL3* | *NUP133* |  |  |  | *STX4* |  |  |
| *NKD1* | *NXNL2* |  |  |  | *STX7* |  |  |
| *NLGN3* | *OGFOD2* |  |  |  | *SULT4A1* |  |  |
| *NMRAL1* | *OGT* |  |  |  | *SUSD5* |  |  |
| *NNMT* | *OLFM1* |  |  |  | *SV2A* |  |  |
| *NOTCH2* | *OSTM1* |  |  |  | *SYNDIG1L* |  |  |
| *NPTX2* | *P3H2* |  |  |  | *SYNE3* |  |  |
| *NR1D1* | *PAFAH1B2* |  |  |  | *SYNGR1* |  |  |
| *NR1H3* | *PAIP2B* |  |  |  | *SYNGR2* |  |  |
| *NR1I2* | *PANK4* |  |  |  | *TACC1* |  |  |
| *NR2F2* | *PANX1* |  |  |  | *TADA3* |  |  |
| *NR4A1* | *PAOX* |  |  |  | *TAF6* |  |  |
| *NRBP1* | *PAQR3* |  |  |  | *TBC1D31* |  |  |
| *NRN1L* | *PAX2* |  |  |  | *TBC1D32* |  |  |
| *NRP1* | *PCDH17* |  |  |  | *TCP11L1* |  |  |
| *NRSN2* | *PCGF5* |  |  |  | *TEDC1* |  |  |
| *NUAK1* | *PCGF6* |  |  |  | *TENT5B* |  |  |
| *NUCB1* | *PCSK5* |  |  |  | *TFAP2B* |  |  |
| *NUDT7* | *PDCD1* |  |  |  | *TGFB1I1* |  |  |
| miR-361-3p | **miR-322-5p** | **miR-365-2-5p** | **miR-1247-3p** | **miR-222-5p** | **Novel 137** | **Novel 141** | **Novel 102** |
| *ODF1* | *PDHB* |  |  |  | *THEMIS2* |  |  |
| *OGDH* | *PDIA6* |  |  |  | *THOC5* |  |  |
| *OLFM1* | *PDK2* |  |  |  | *THRAP3* |  |  |
| *OLFML2A* | *PDRG1* |  |  |  | *THRB* |  |  |
| *OPRL1* | *PDXK* |  |  |  | *TIGAR* |  |  |
| *OSBPL5* | *PDZD7* |  |  |  | *TIMD4* |  |  |
| *OSGIN2* | *PDZD8* |  |  |  | *TIMP4* |  |  |
| *OTOA* | *PEAK1* |  |  |  | *TLK2* |  |  |
| *OXSR1* | *PELI3* |  |  |  | *TM4SF4* |  |  |
| *OXT* | *PEX16* |  |  |  | *TMCC2* |  |  |
| *P2RX3* | *PEX19* |  |  |  | *TMEM127* |  |  |
| *P3H1* | *PEX5* |  |  |  | *TMEM131* |  |  |
| *P3H2* | *PGAP3* |  |  |  | *TMEM241* |  |  |
| *PACSIN1* | *PHF13* |  |  |  | *TMEM26* |  |  |
| *PADI2* | *PHF14* |  |  |  | *TMEM63C* |  |  |
| *PAIP2B* | *PHF6* |  |  |  | *TMEM86A* |  |  |
| *PAK4* | *PHKG2* |  |  |  | *TNFAIP3* |  |  |
| *PALM* | *PHLDB1* |  |  |  | *TNFSF9* |  |  |
| *PANX1* | *PI16* |  |  |  | *TNMD* |  |  |
| *PANX2* | *PI4KB* |  |  |  | *TNRC18* |  |  |
| *PAX1* | *PID1* |  |  |  | *TOPBP1* |  |  |
| *PAX2* | *PIGB* |  |  |  | *TPI1* |  |  |
| *PAX3* | *PISD* |  |  |  | *TPM3* |  |  |
| *PCGF2* | *PIWIL1* |  |  |  | *TPRA1* |  |  |
| *PCGF3* | *PLA1A* |  |  |  | *TRARG1* |  |  |
| *PCNA* | *PLEKHA8* |  |  |  | *TRIM41* |  |  |
| *PCSK6* | *PLEKHD1* |  |  |  | *TRIM46* |  |  |
| *PDCD1* | *PLPBP* |  |  |  | *TRIML1* |  |  |
| *PDE6C* | *PLPP4* |  |  |  | *TSEN15* |  |  |
| *PDE8A* | *PLXNA1* |  |  |  | *TSPAN14* |  |  |
| *PDGFRB* | *PLXNA2* |  |  |  | *TSPAN33* |  |  |
| *PDK2* | *PLXNB1* |  |  |  | *TTC13* |  |  |
| *PDRG1* | *PNMA3* |  |  |  | *TTC7A* |  |  |
| *PDX1* | *PNP* |  |  |  | *TTLL12* |  |  |
| *PDXK* | *POFUT1* |  |  |  | *TUB* |  |  |
| *PDZD4* | *POLM* |  |  |  | *TUBB* |  |  |
| *PDZD7* | *POLR1E* |  |  |  | *TUBE1* |  |  |
| *PEG3* | *POLR2D* |  |  |  | *UBE2B* |  |  |
| *PELI3* | *POLR2H* |  |  |  | *UBE2J2* |  |  |
| *PEPD* | *POLR3C* |  |  |  | *UBE2M* |  |  |
| miR-361-3p | **miR-322-5p** | **miR-365-2-5p** | **miR-1247-3p** | **miR-222-5p** | **Novel 137** | **Novel 141** | **Novel 102** |
| *PEX10* | *POLR3D* |  |  |  | *UBE2Q2* |  |  |
| *PEX14* | *PPARG* |  |  |  | *UBR3* |  |  |
| *PEX16* | *PPAT* |  |  |  | *UBTD2* |  |  |
| *PGAP3* | *PPCDC* |  |  |  | *UCP3* |  |  |
| *PHC2* | *PPFIA2* |  |  |  | *UFD1* |  |  |
| *PHF1* | *PPHLN1* |  |  |  | *UHRF1BP1* |  |  |
| *PHF6* | *PPIH* |  |  |  | *USB1* |  |  |
| *PHGDH* | *PPIL1* |  |  |  | *USH1G* |  |  |
| *PHLDB1* | *PPP1R16A* |  |  |  | *USP31* |  |  |
| *PHOSPHO1* | *PPP2R2D* |  |  |  | *UST* |  |  |
| *PI4KB* | *PPP2R5E* |  |  |  | *VAX1* |  |  |
| *PIANP* | *PPP6C* |  |  |  | *VDR* |  |  |
| *PIAS3* | *PPP6R2* |  |  |  | *WBP1L* |  |  |
| *PID1* | *PPP6R3* |  |  |  | *WDR18* |  |  |
| *PIP4K2B* | *PPT2* |  |  |  | *WDR46* |  |  |
| *PIWIL1* | *PPTC7* |  |  |  | *WDR47* |  |  |
| *PKNOX2* | *PRICKLE1* |  |  |  | *WDR59* |  |  |
| *PLA2G2F* | *PRKAB2* |  |  |  | *WDR63* |  |  |
| *PLA2G3* | *PRKCB* |  |  |  | *WNT16* |  |  |
| *PLAGL2* | *PRR14L* |  |  |  | *WNT2B* |  |  |
| *PLEKHA6* | *PSAT1* |  |  |  | *WNT4* |  |  |
| *PLEKHB1* | *PSIP1* |  |  |  | *WNT5B* |  |  |
| *PLEKHG5* | *PSKH1* |  |  |  | *WNT8A* |  |  |
| *PLEKHM2* | *PSME3* |  |  |  | *WNT9A* |  |  |
| *PLXNA1* | *PSME4* |  |  |  | *XPO6* |  |  |
| *PLXNB1* | *PTCD2* |  |  |  | *XXYLT1* |  |  |
| *PNKD* | *PTCH1* |  |  |  | *ZCCHC2* |  |  |
| *PNMA3* | *PTGES3L-AARSD1* | |  |  | *ZER1* |  |  |
| *POFUT1* | *PTK2B* |  |  |  | *ZIC2* |  |  |
| *POLDIP2* | *PWWP2B* |  |  |  | *ZMAT4* |  |  |
| *POLM* | *PXDC1* |  |  |  | *ZMYND10* |  |  |
| *POLR1E* | *PXDNL* |  |  |  | *ZMYND8* |  |  |
| *POLR2B* | *QDPR* |  |  |  | *ZNF133* |  |  |
| *POLR2C* | *RAC2* |  |  |  | *ZNF48* |  |  |
| *POLR3D* | *RACGAP1* |  |  |  | *ZNF507* |  |  |
| *PPARD* | *RALGAPA2* |  |  |  | *ZNF512* |  |  |
| *PPARGC1A* | *RALGPS2* |  |  |  | *ZNF532* |  |  |
| *PPIF* | *RANBP3* |  |  |  | *ZNF629* |  |  |
| *PPIL2* | *RAP1GAP2* |  |  |  | *ZNF672* |  |  |
| *PPIP5K1* | *RASD2* |  |  |  | *ZNF768* |  |  |
| miR-361-3p | **miR-322-5p** | **miR-365-2-5p** | **miR-1247-3p** | **miR-222-5p** | **Novel 137** | **Novel 141** | **Novel 102** |
| *PPP1R16A* | *RASEF* |  |  |  | *ZNF862* |  |  |
| *PPP1R16B* | *RASGEF1B* |  |  |  | *ZP1* |  |  |
| *PPP1R8* | *RASGRP2* |  |  |  | *ZSCAN22* |  |  |
| *PPP1R9B* | *RASSF2* |  |  |  | *ZSWIM8* |  |  |
| *PPP2R2D* | *RASSF5* |  |  |  | *ZWINT* |  |  |
| *PPP2R5B* | *RASSF8* |  |  |  |  |  |  |
| *PPP3CA* | *RBCK1* |  |  |  |  |  |  |
| *PPP6C* | *RBM23* |  |  |  |  |  |  |
| *PPTC7* | *RBM24* |  |  |  |  |  |  |
| *PQLC2* | *RBM39* |  |  |  |  |  |  |
| *PRDM2* | *RBM6* |  |  |  |  |  |  |
| *PREB* | *RCOR3* |  |  |  |  |  |  |
| *PRKCG* | *REEP1* |  |  |  |  |  |  |
| *PRKD3* | *REEP3* |  |  |  |  |  |  |
| *PRR5* | *REPIN1* |  |  |  |  |  |  |
| *PRUNE1* | *RET* |  |  |  |  |  |  |
| *PSD4* | *RFWD3* |  |  |  |  |  |  |
| *PSME4* | *RFX3* |  |  |  |  |  |  |
| *PSMF1* | *RGMA* |  |  |  |  |  |  |
| *PTDSS2* | *RIC8A* |  |  |  |  |  |  |
| *PTGER2* | *RIMKLA* |  |  |  |  |  |  |
| *PTTG1IP* | *RMI2* |  |  |  |  |  |  |
| *PWWP2B* | *RMND5B* |  |  |  |  |  |  |
| *PXDC1* | *RNF111* |  |  |  |  |  |  |
| *PYGO2* | *RNF185* |  |  |  |  |  |  |
| *QDPR* | *RNF20* |  |  |  |  |  |  |
| *R3HDM2* | *RNF43* |  |  |  |  |  |  |
| *R3HDM4* | *ROGDI* |  |  |  |  |  |  |
| *RALGAPA2* | *RPL15* |  |  |  |  |  |  |
| *RAP1GAP* | *RPL7A* |  |  |  |  |  |  |
| *RAP1GAP2* | *RPP30* |  |  |  |  |  |  |
| *RASGRP2* | *RPRD1B* |  |  |  |  |  |  |
| *RASSF2* | *RPS6KA3* |  |  |  |  |  |  |
| *RASSF8* | *RPTOR* |  |  |  |  |  |  |
| *RAX* | *RRM2B* |  |  |  |  |  |  |
| *RBMX* | *RRP1* |  |  |  |  |  |  |
| *RCC1* | *RS1* |  |  |  |  |  |  |
| *RDH16* | *RSBN1* |  |  |  |  |  |  |
| *REEP2* | *RSPO2* |  |  |  |  |  |  |
| *REEP3* | *RUBCN* |  |  |  |  |  |  |
| miR-361-3p | **miR-322-5p** | **miR-365-2-5p** | **miR-1247-3p** | **miR-222-5p** | **Novel 137** | **Novel 141** | **Novel 102** |
| *REN* | *RUBCNL* |  |  |  |  |  |  |
| *REPIN1* | *RUFY1* |  |  |  |  |  |  |
| *REPS2* | *RWDD1* |  |  |  |  |  |  |
| *RFC5* | *RWDD4* |  |  |  |  |  |  |
| *RFNG* | *S100B* |  |  |  |  |  |  |
| *RGL1* | *SALL1* |  |  |  |  |  |  |
| *RGMB* | *SALL4* |  |  |  |  |  |  |
| *RHBDD1* | *SAMD10* |  |  |  |  |  |  |
| *RIC8A* | *SCGB1A1* |  |  |  |  |  |  |
| *RIC8B* | *SCN3A* |  |  |  |  |  |  |
| *RIMS4* | *SDC1* |  |  |  |  |  |  |
| *RNF185* | *SDCBP* |  |  |  |  |  |  |
| *RNF20* | *SDCCAG8* |  |  |  |  |  |  |
| *RNF215* | *SEH1L* |  |  |  |  |  |  |
| *RNF32* | *SELENOS* |  |  |  |  |  |  |
| *RNF43* | *SEMA3D* |  |  |  |  |  |  |
| *RNPEPL1* | *SEMA6D* |  |  |  |  |  |  |
| *ROGDI* | *SENP2* |  |  |  |  |  |  |
| *ROM1* | *SERBP1* |  |  |  |  |  |  |
| *RPL8* | *SERPINB12* |  |  |  |  |  |  |
| *RPRD2* | *SFPQ* |  |  |  |  |  |  |
| *RPS19BP1* | *SFRP1* |  |  |  |  |  |  |
| *RPTOR* | *SFXN2* |  |  |  |  |  |  |
| *RRBP1* | *SFXN3* |  |  |  |  |  |  |
| *RS1* | *SGK1* |  |  |  |  |  |  |
| *RSPO1* | *SGTB* |  |  |  |  |  |  |
| *RWDD4* | *SH3BP4* |  |  |  |  |  |  |
| *RXRA* | *SH3KBP1* |  |  |  |  |  |  |
| *S100A16* | *SH3PXD2B* |  |  |  |  |  |  |
| *SAMD10* | *SHCBP1* |  |  |  |  |  |  |
| *SAMD14* | *SHOX* |  |  |  |  |  |  |
| *SAMD5* | *SHROOM3* |  |  |  |  |  |  |
| *SCAMP4* | *SIAH1* |  |  |  |  |  |  |
| *SCARA3* | *SIDT1* |  |  |  |  |  |  |
| *SCARB1* | *SIDT2* |  |  |  |  |  |  |
| *SCN1B* | *SIX4* |  |  |  |  |  |  |
| *SDC1* | *SKI* |  |  |  |  |  |  |
| *SDHB* | *SLC10A6* |  |  |  |  |  |  |
| *SEC61A1* | *SLC12A2* |  |  |  |  |  |  |
| *SEMA4F* | *SLC13A3* |  |  |  |  |  |  |
| *SEMA5A* | *SLC20A2* |  |  |  |  |  |  |
| miR-361-3p | **miR-322-5p** | **miR-365-2-5p** | **miR-1247-3p** | **miR-222-5p** | **Novel 137** | **Novel 141** | **Novel 102** |
| *SEMA7A* | *SLC22A17* |  |  |  |  |  |  |
| *SENP2* | *SLC25A30* |  |  |  |  |  |  |
| *SEPTIN9* | *SLC25A6* |  |  |  |  |  |  |
| *SERTAD4* | *SLC26A4* |  |  |  |  |  |  |
| *SF3A1* | *SLC29A2* |  |  |  |  |  |  |
| *SF3B3* | *SLC35G1* |  |  |  |  |  |  |
| *SFPQ* | *SLC37A1* |  |  |  |  |  |  |
| *SFRP5* | *SLC38A3* |  |  |  |  |  |  |
| *SFXN1* | *SLC39A14* |  |  |  |  |  |  |
| *SGCG* | *SLC39A3* |  |  |  |  |  |  |
| *SGSM3* | *SLC39A8* |  |  |  |  |  |  |
| *SGTA* | *SLC41A1* |  |  |  |  |  |  |
| *SH2B1* | *SLC41A2* |  |  |  |  |  |  |
| *SH2D5* | *SLC43A2* |  |  |  |  |  |  |
| *SH2D7* | *SLC4A3* |  |  |  |  |  |  |
| *SH3BP4* | *SLC6A15* |  |  |  |  |  |  |
| *SH3KBP1* | *SLC9A3R2* |  |  |  |  |  |  |
| *SH3PXD2B* | *SLC9A4* |  |  |  |  |  |  |
| *SH3RF2* | *SLC9A9* |  |  |  |  |  |  |
| *SHISA4* | *SLCO2A1* |  |  |  |  |  |  |
| *SHOX* | *SLCO2B1* |  |  |  |  |  |  |
| *SHROOM1* | *SLK* |  |  |  |  |  |  |
| *SIDT2* | *SLMAP* |  |  |  |  |  |  |
| *SKI* | *SMAD3* |  |  |  |  |  |  |
| *SLC13A1* | *SMAD4* |  |  |  |  |  |  |
| *SLC15A1* | *SMIM20* |  |  |  |  |  |  |
| *SLC17A9* | *SMPX* |  |  |  |  |  |  |
| *SLC25A25* | *SMUG1* |  |  |  |  |  |  |
| *SLC25A45* | *SMURF1* |  |  |  |  |  |  |
| *SLC25A6* | *SNAP25* |  |  |  |  |  |  |
| *SLC29A3* | *SNAPC5* |  |  |  |  |  |  |
| *SLC2A4* | *SNCG* |  |  |  |  |  |  |
| *SLC35F1* | *SNRK* |  |  |  |  |  |  |
| *SLC37A1* | *SNTB2* |  |  |  |  |  |  |
| *SLC38A8* | *SNX21* |  |  |  |  |  |  |
| *SLC39A3* | *SNX33* |  |  |  |  |  |  |
| *SLC41A1* | *SORT1* |  |  |  |  |  |  |
| *SLC45A3* | *SOX9* |  |  |  |  |  |  |
| *SLC48A1* | *SPATA19* |  |  |  |  |  |  |
| *SLC5A1* | *SPRYD3* |  |  |  |  |  |  |
| miR-361-3p | **miR-322-5p** | **miR-365-2-5p** | **miR-1247-3p** | **miR-222-5p** | **Novel 137** | **Novel 141** | **Novel 102** |
| *SLC6A1* | *SRP72* |  |  |  |  |  |  |
| *SLC6A14* | *SRPK1* |  |  |  |  |  |  |
| *SLC6A2* | *SSFA2* |  |  |  |  |  |  |
| *SLC7A6* | *SSR1* |  |  |  |  |  |  |
| *SLC7A6OS* | *ST7L* |  |  |  |  |  |  |
| *SLC9A3R2* | *STARD3* |  |  |  |  |  |  |
| *SLC9A5* | *STARD8* |  |  |  |  |  |  |
| *SMAD5* | *STAT6* |  |  |  |  |  |  |
| *SMC1B* | *STK10* |  |  |  |  |  |  |
| *SMPD3* | *STX1A* |  |  |  |  |  |  |
| *SMTN* | *STX7* |  |  |  |  |  |  |
| *SNTB1* | *STYX* |  |  |  |  |  |  |
| *SNX18* | *SUSD6* |  |  |  |  |  |  |
| *SP7* | *SV2C* |  |  |  |  |  |  |
| *SPATA2L* | *SYNE3* |  |  |  |  |  |  |
| *SPATA45* | *SYNE4* |  |  |  |  |  |  |
| *SPOPL* | *SYNJ1* |  |  |  |  |  |  |
| *SRD5A1* | *SYNPO2L* |  |  |  |  |  |  |
| *SRD5A2* | *SYT3* |  |  |  |  |  |  |
| *SREBF1* | *SYT4* |  |  |  |  |  |  |
| *SRI* | *TACC1* |  |  |  |  |  |  |
| *STC2* | *TACO1* |  |  |  |  |  |  |
| *STK24* | *TAF13* |  |  |  |  |  |  |
| *STK40* | *TAF4B* |  |  |  |  |  |  |
| *STRIP1* | *TANGO6* |  |  |  |  |  |  |
| *STRN4* | *TAX1BP3* |  |  |  |  |  |  |
| *STX11* | *TBC1D14* |  |  |  |  |  |  |
| *STX1A* | *TBC1D19* |  |  |  |  |  |  |
| *STX5* | *TBC1D9* |  |  |  |  |  |  |
| *SUSD5* | *TBCCD1* |  |  |  |  |  |  |
| *SUSD6* | *TBL1XR1* |  |  |  |  |  |  |
| *SV2A* | *TBL2* |  |  |  |  |  |  |
| *SV2B* | *TBPL1* |  |  |  |  |  |  |
| *SVOP* | *TBX21* |  |  |  |  |  |  |
| *SYNDIG1L* | *TCN2* |  |  |  |  |  |  |
| *SYNE3* | *TCP11L1* |  |  |  |  |  |  |
| *SYNE4* | *TCP11L2* |  |  |  |  |  |  |
| *SYNGR1* | *TDRP* |  |  |  |  |  |  |
| *SYT3* | *TEDC1* |  |  |  |  |  |  |
| *SYVN1* | *TFCP2* |  |  |  |  |  |  |
| miR-361-3p | **miR-322-5p** | **miR-365-2-5p** | **miR-1247-3p** | **miR-222-5p** | **Novel 137** | **Novel 141** | **Novel 102** |
| *TACC1* | *TGFBR3* |  |  |  |  |  |  |
| *TACR2* | *THEMIS2* |  |  |  |  |  |  |
| *TADA3* | *THOP1* |  |  |  |  |  |  |
| *TAF5L* | *THRAP3* |  |  |  |  |  |  |
| *TAF6* | *TIAL1* |  |  |  |  |  |  |
| *TAGLN* | *TIGAR* |  |  |  |  |  |  |
| *TAPT1* | *TJAP1* |  |  |  |  |  |  |
| *TAZ* | *TKFC* |  |  |  |  |  |  |
| *TBC1D14* | *TKTL1* |  |  |  |  |  |  |
| *TBC1D5* | *TLCD2* |  |  |  |  |  |  |
| *TBRG4* | *TLE4* |  |  |  |  |  |  |
| *TEDC1* | *TMEM108* |  |  |  |  |  |  |
| *TENT5B* | *TMEM127* |  |  |  |  |  |  |
| *TFE3* | *TMEM150A* |  |  |  |  |  |  |
| *TGFBR3* | *TMEM179* |  |  |  |  |  |  |
| *THAP10* | *TMEM198* |  |  |  |  |  |  |
| *THRA* | *TMEM255A* |  |  |  |  |  |  |
| *THRAP3* | *TMEM26* |  |  |  |  |  |  |
| *THRB* | *TMEM37* |  |  |  |  |  |  |
| *THTPA* | *TMEM72* |  |  |  |  |  |  |
| *TIMM13* | *TMEM87A* |  |  |  |  |  |  |
| *TIMP4* | *TMPRSS13* |  |  |  |  |  |  |
| *TLCD2* | *TMX2* |  |  |  |  |  |  |
| *TLDC1* | *TMX3* |  |  |  |  |  |  |
| *TLE2* | *TNFAIP1* |  |  |  |  |  |  |
| *TMBIM1* | *TNIP2* |  |  |  |  |  |  |
| *TMCO4* | *TOP2B* |  |  |  |  |  |  |
| *TMEM106A* | *TOP3A* |  |  |  |  |  |  |
| *TMEM120A* | *TOPORS* |  |  |  |  |  |  |
| *TMEM136* | *TOR1B* |  |  |  |  |  |  |
| *TMEM17* | *TPI1* |  |  |  |  |  |  |
| *TMEM175* | *TRABD2B* |  |  |  |  |  |  |
| *TMEM179* | *TRADD* |  |  |  |  |  |  |
| *TMEM198* | *TRAT1* |  |  |  |  |  |  |
| *TMEM214* | *TRIM2* |  |  |  |  |  |  |
| *TMEM248* | *TRIM37* |  |  |  |  |  |  |
| *TMEM47* | *TRIM46* |  |  |  |  |  |  |
| *TMEM52B* | *TRIP11* |  |  |  |  |  |  |
| *TMEM63C* | *TSPAN14* |  |  |  |  |  |  |
| *TMEM86A* | *TSPAN33* |  |  |  |  |  |  |
| miR-361-3p | **miR-322-5p** | **miR-365-2-5p** | **miR-1247-3p** | **miR-222-5p** | **Novel 137** | **Novel 141** | **Novel 102** |
| *TNFAIP3* | *TSPEAR* |  |  |  |  |  |  |
| *TNS1* | *TSPYL2* |  |  |  |  |  |  |
| *TOLLIP* | *TTC1* |  |  |  |  |  |  |
| *TOP2B* | *TTC21B* |  |  |  |  |  |  |
| *TOPBP1* | *TTL* |  |  |  |  |  |  |
| *TPH1* | *TUBB1* |  |  |  |  |  |  |
| *TPI1* | *TUBB6* |  |  |  |  |  |  |
| *TPRA1* | *TUT7* |  |  |  |  |  |  |
| *TRABD* | *TXNDC16* |  |  |  |  |  |  |
| *TRAF4* | *TXNIP* |  |  |  |  |  |  |
| *TRAFD1* | *TYRO3* |  |  |  |  |  |  |
| *TRIM11* | *TYW3* |  |  |  |  |  |  |
| *TRIM33* | *U2SURP* |  |  |  |  |  |  |
| *TRIM36* | *UBE2B* |  |  |  |  |  |  |
| *TRPV3* | *UBE2J1* |  |  |  |  |  |  |
| *TSPAN14* | *UBE2K* |  |  |  |  |  |  |
| *TSPYL2* | *UBE2QL1* |  |  |  |  |  |  |
| *TTC13* | *UBE2V1* |  |  |  |  |  |  |
| *TTL* | *UBE3D* |  |  |  |  |  |  |
| *TTLL12* | *UBFD1* |  |  |  |  |  |  |
| *TXNDC16* | *UBIAD1* |  |  |  |  |  |  |
| *TYW3* | *UBP1* |  |  |  |  |  |  |
| *UBALD2* | *UBR3* |  |  |  |  |  |  |
| *UBASH3A* | *UCP3* |  |  |  |  |  |  |
| *UBE2V1* | *UHRF1BP1* |  |  |  |  |  |  |
| *UBP1* | *ULK3* |  |  |  |  |  |  |
| *UCP3* | *UNC119B* |  |  |  |  |  |  |
| *UHRF1BP1* | *UPK3A* |  |  |  |  |  |  |
| *UNC119B* | *UROC1* |  |  |  |  |  |  |
| *UNC5CL* | *USP12* |  |  |  |  |  |  |
| *UPK3A* | *USP14* |  |  |  |  |  |  |
| *UROC1* | *USP20* |  |  |  |  |  |  |
| *USB1* | *USP22* |  |  |  |  |  |  |
| *USH1G* | *USP31* |  |  |  |  |  |  |
| *USP11* | *USP39* |  |  |  |  |  |  |
| *USP20* | *USP44* |  |  |  |  |  |  |
| *USP22* | *USP7* |  |  |  |  |  |  |
| *USP46* | *VAMP8* |  |  |  |  |  |  |
| *USP47* | *VDR* |  |  |  |  |  |  |
| *USP54* | *VIPR1* |  |  |  |  |  |  |
| *USP7* | *VIT* |  |  |  |  |  |  |
| miR-361-3p | **miR-322-5p** | **miR-365-2-5p** | **miR-1247-3p** | **miR-222-5p** | **Novel 137** | **Novel 141** | **Novel 102** |
| *VASH1* | *VPS36* |  |  |  |  |  |  |
| *VAV3* | *VSTM2A* |  |  |  |  |  |  |
| *VPS26C* | *VWA7* |  |  |  |  |  |  |
| *VSTM2L* | *WASF2* |  |  |  |  |  |  |
| *VSTM5* | *WBP1L* |  |  |  |  |  |  |
| *VWA7* | *WBP2* |  |  |  |  |  |  |
| *VXN* | *WDR18* |  |  |  |  |  |  |
| *WASF2* | *WDR20* |  |  |  |  |  |  |
| *WBP1L* | *WDR33* |  |  |  |  |  |  |
| *WBP2* | *WDR47* |  |  |  |  |  |  |
| *WDR13* | *WDR62* |  |  |  |  |  |  |
| *WDR18* | *WDR7* |  |  |  |  |  |  |
| *WDR34* | *WDSUB1* |  |  |  |  |  |  |
| *WDR45B* | *WEE1* |  |  |  |  |  |  |
| *WDR5* | *WISP1* |  |  |  |  |  |  |
| *WDR63* | *WNT4* |  |  |  |  |  |  |
| *WDR7* | *WNT7A* |  |  |  |  |  |  |
| *WNT2* | *WNT8A* |  |  |  |  |  |  |
| *WNT4* | *WNT9A* |  |  |  |  |  |  |
| *WNT9A* | *WWC1* |  |  |  |  |  |  |
| *WSB1* | *WWP2* |  |  |  |  |  |  |
| *WWP2* | *XIRP2* |  |  |  |  |  |  |
| *XPO7* | *XPO6* |  |  |  |  |  |  |
| *YPEL2* | *XPO7* |  |  |  |  |  |  |
| *YPEL4* | *YME1L1* |  |  |  |  |  |  |
| *YY1* | *YPEL2* |  |  |  |  |  |  |
| *ZBTB47* | *YPEL4* |  |  |  |  |  |  |
| *ZC3H7B* | *YTHDC1* |  |  |  |  |  |  |
| *ZDHHC5* | *YWHAQ* |  |  |  |  |  |  |
| *ZDHHC8* | *YY1* |  |  |  |  |  |  |
| *ZER1* | *ZBTB10* |  |  |  |  |  |  |
| *ZFHX3* | *ZBTB47* |  |  |  |  |  |  |
| *ZFP1* | *ZC3H12B* |  |  |  |  |  |  |
| *ZIC2* | *ZCCHC2* |  |  |  |  |  |  |
| *ZIC3* | *ZCCHC24* |  |  |  |  |  |  |
| *ZKSCAN8* | *ZDHHC15* |  |  |  |  |  |  |
| *ZNF182* | *ZDHHC16* |  |  |  |  |  |  |
| *ZNF236* | *ZFHX3* |  |  |  |  |  |  |
| *ZNF397* | *ZFHX4* |  |  |  |  |  |  |
| *ZNF423* | *ZFYVE1* |  |  |  |  |  |  |
| *ZNF467* | *ZIC1* |  |  |  |  |  |  |
| miR-361-3p | **miR-322-5p** | **miR-365-2-5p** | **miR-1247-3p** | **miR-222-5p** | **Novel 137** | **Novel 141** | **Novel 102** |
| *ZNF513* | *ZMYM2* |  |  |  |  |  |  |
| *ZNF550* | *ZNF133* |  |  |  |  |  |  |
| *ZNF598* | *ZNF25* |  |  |  |  |  |  |
| *ZNF629* | *ZNF326* |  |  |  |  |  |  |
| *ZNF787* | *ZNF341* |  |  |  |  |  |  |
| *ZP1* | *ZNF350* |  |  |  |  |  |  |
| *ZSCAN20* | *ZNF397* |  |  |  |  |  |  |
| *ZSWIM9* | *ZNF438* |  |  |  |  |  |  |
| *ZWINT* | *ZNF449* |  |  |  |  |  |  |
|  | *ZNF502* |  |  |  |  |  |  |
|  | *ZNF512* |  |  |  |  |  |  |
|  | *ZNF672* |  |  |  |  |  |  |
|  | *ZNF772* |  |  |  |  |  |  |
|  | *ZNF787* |  |  |  |  |  |  |
|  | *ZNF862* |  |  |  |  |  |  |
|  | *ZPBP* |  |  |  |  |  |  |
