# Supplementary material for: Detection and characterization of microRNA expression profiling and its target genes in response to canine parvovirus in Crandell Reese Feline Kidney cells
Source: PeerJ. 2020 Feb 12;8:e8522. doi: 10.7717/peerj.8522 (PMC7023829; doi:10.7717/peerj.8522)
Supplement: Supplemental Information 8 [file peerj-08-8522-s008.docx]

**Supplementary Table 8 List of significant differently expressed miRNAs, prediction of number of target genes, and number of related gene ontology (GO) functional analysis identified in the current study.**

|  |  | **Gene Ontology (GO)** | | |
| --- | --- | --- | --- | --- |
| **miRNA** | **No of target (*n*)** | **Biological Process (BP)** | **Molecular Function (MF)** | **Cellular component (CC)** |
| miR-361 | 965 | 77 | 18 | 1 |
| miR-322 | 972 | 88 | 13 | 33 |
| miR-365 | 437 | 20 | 0 | 0 |
| miR-1247 | 229 | 33 | 5 | 0 |
| miR-222 | 357 | 0 | 0 | 2 |
| Novel-137 | 678 | 53 | 7 | 6 |
| Novel-141 | 282 | 0 | 1 | 0 |
| Novel-102 | 236 | 8 | 0 | 0 |
