# Supplementary figures and images for: Detection and characterization of microRNA expression profiling and its target genes in response to canine parvovirus in Crandell Reese Feline Kidney cells
Source: PeerJ. 2020 Feb 12;8:e8522. doi: 10.7717/peerj.8522 (PMC7023829; doi:10.7717/peerj.8522)

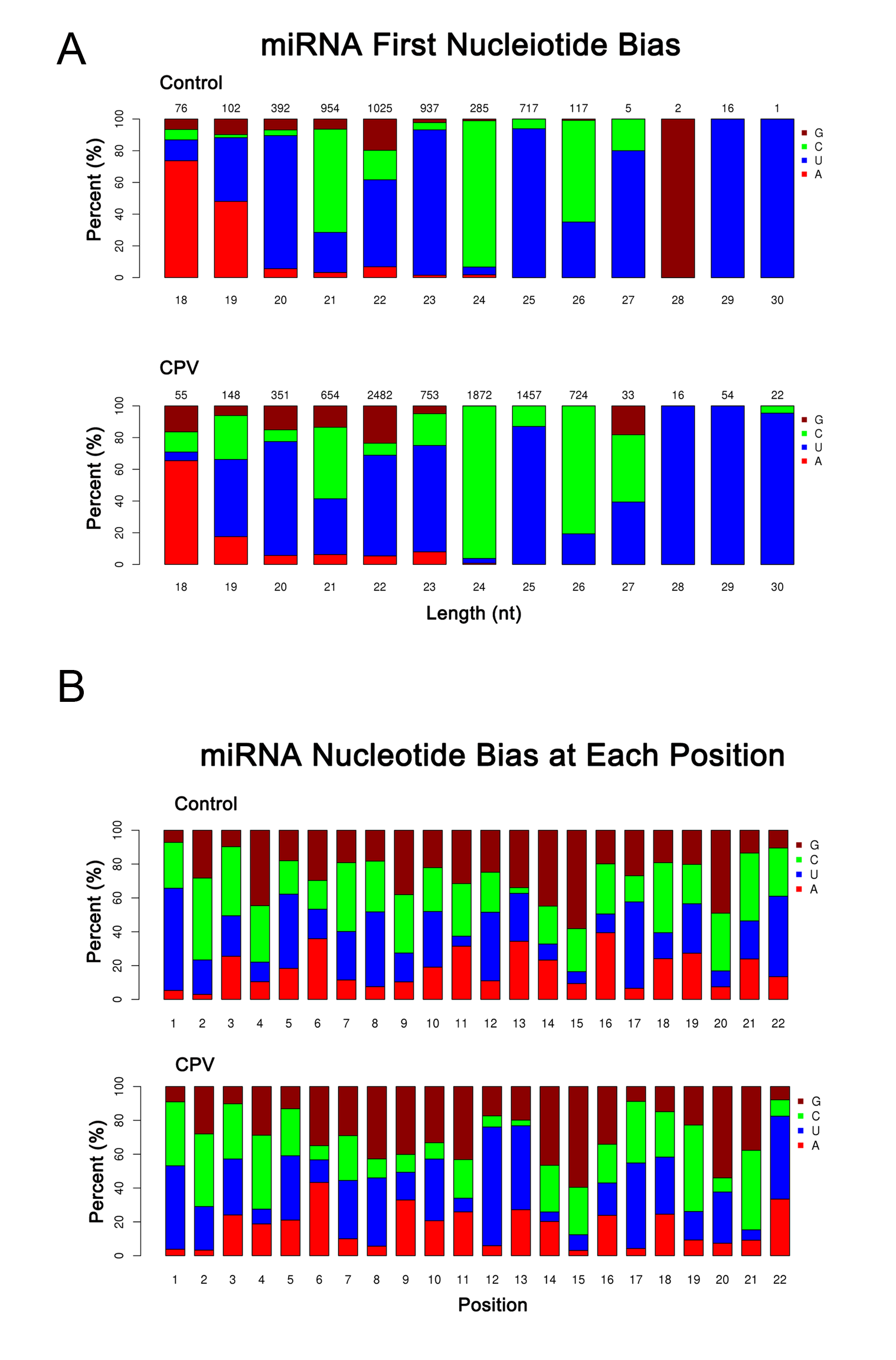

Supplement: Supplemental Information 9 — (A) First nucleotide bias of novel F. catus miRNA candidates in Control and CPV libraries. The number on top of the bars indicated the number of sequences corresponding to the miRNA length (nt). x-axis, length of miRNAs; y-axis, responding percent of each nucleotide. (B) Nucleotide bias at each position in Control and CPV libraries. x-axis, Position of nucleotide in miRNAs; y-axis, responding percent of each nucleotide. [file peerj-08-8522-s009.png]

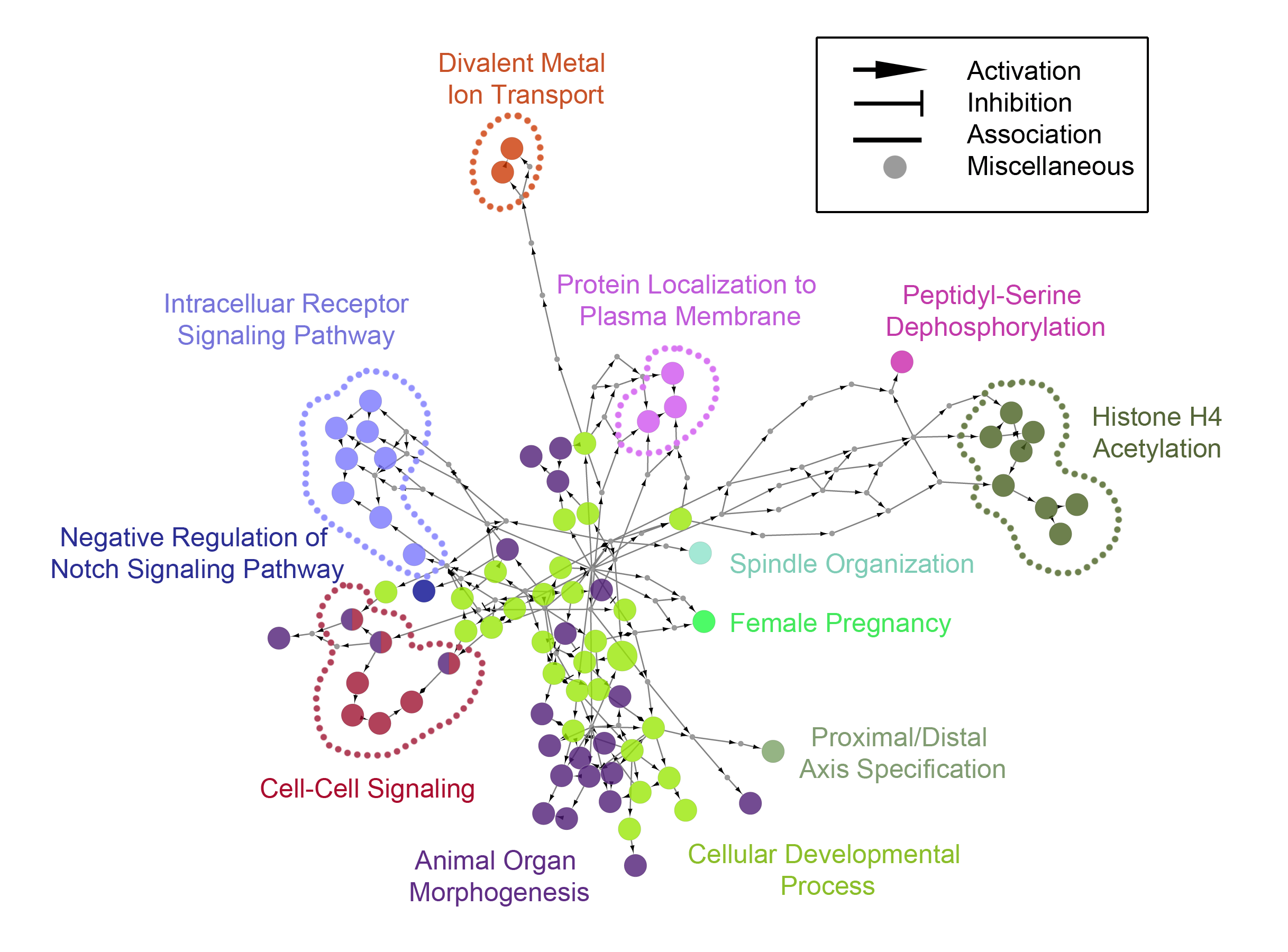

Supplement: Supplemental Information 10 — ClueGO/CluePedia network shows a function group network with GO terms as nodes linked of target genes of Novel 137. The labels of the most significant term per group are shown. The node size depicts the significant enrichment. Functionally related groups (in part) are emphasized by overlapping of colors. [file peerj-08-8522-s010.png]
